# Supplementary material for: GLS-driven glutamine catabolism contributes to prostate cancer radiosensitivity by regulating the redox state, stemness and ATG5-mediated autophagy
Source: Theranostics. 2021 Jun 26;11(16):7844–68. doi: 10.7150/thno.58655 (PMC8315064; doi:10.7150/thno.58655)
Supplement: Supplementary file 1 — Supplementary figures and tables. [file thnov11p7844s1.pdf]

Supplementary information

Table S1. Characteristics of PCa patients whose blood plasma samples were analyzed for amino acid metabolites (n = 20).

Table S2. Analysis of blood plasma metabolites and PSA-DT in PCa patients before and after radiotherapy.

Table S3. Clinical characteristics of PCa patients treated with curatively-intended, definitive radiotherapy at the Department of Radiotherapy and Radiation Oncology, University Hospital Carl Gustav Carus and Faculty of Medicine (n = 74). Formalin-fixed paraffin-embedded (FFPE) tumor tissues from these patients were used for analysis of *MYC* gene expression by nanoString technology.

Table S4. Clinical characteristics of PCa patients (n = 14). Primary prostate cancer and adjacent normal tissues (benign hyperplasia, BPH) from these patients were used for primary tissue cultures and radiobiological colony forming assays.

Table S5. Primers, siRNA oligos and antibodies used in the study.

Table S6. Targeted metabolomics of amino acids and biogenic amines.

Table S7. Concentration of metabolites used in for metabolic flux analysis. Concentrations are reported as µM/500k cells (Average ± Standard Error).

Table S1. Characteristics of PCa patients whose blood plasma samples were analyzed for amino acid metabolites

**Study ID**	1st PSA pre RT	2nd PSA pre RT	**PSA-DT pre RT**	1st PSA after RT	2nd PSA after RT	**PSA-DT post RT**	TNM	Gleason	Therapy
**HT1**	0.19	2.57	1.63	0.64	0.10	-5.05	pT3,pN1	4+4	ADT + local RT
**HT2**	21.10	126.00	12.06				cT3 cN1 cM1	5+4	ADT + local RT
**HT3**	10.30	18.10	1.72	13.80	50.30	1.48	pT3, pN0	4+5	ADT + local RT
**HT4**	50.30	67.40	0.95	48.20	81.30	0.31	cT4, cN1, cM1	5+5	ADT + local RT
**HT6**	2.47	4.40	4.84	0.90	3.5	8.93	pT2,pN0,pM0	3+4	ADT + local RT
**HT7**	0.18	4.00	1.34				pT3,pN1,cM0	4+4	ADT + local RT
**HT8**	12.40	24.40	6.59	0.80	1	73.72	pT2,pN0,pM0	3+3	ADT + local RT
**HT9**	1.6	2.20	4.79	0.06	0.13	7.14	pT2,pN0,pM0		ADT + local RT
**HT10**	5.90	7.10	8.36	0.10	0.11	78.54	pT3,pN1,cM0		ADT + local RT
**HT11**	1.00	8.00	2.57	21.40	43.20	1.05	pT3,pN1	4+4	ADT + local RT
**HT12**	5.70	13.00	1.71	2.2	11.00	3.27	M1		ADT + local RT
**HT13**	0.9	1.50	8.91	3.10	3.15	31.77	pT1,pN0	3+3	ADT + local RT
**HT15**	0.17	2.30	4.10	0.10	0.01	-2.08	pT3a, pN0	4+4	ADT + local RT
**HT16**	2.00	4.26	3.09	0.05	13.00	1.18	pT2,pN1,Mo	3+4	ADT + local RT
**HT17**	2.80	4.75	3.98	0.06	0.70	1.75	pT3b, pN0, cM0	4+5	ADT + local RT
**HT18**	53.00	60.00	4.84	75.00	78.00	105.45	pT2c,pN0	3+4	ADT + local RT
**HT21**	66.00	168.00	3.04	90.00	128.00	7.35	M1	4+5	ADT + local RT
**HT22**	0.03	0.10	6.37	0.05			pT3b,pN1,M0	5+4	ADT + local RT
**HT23**	3.50	4.30	20.43				cT2,cN0,cM0	3+4	ADT + local RT
**HT24**	0.17	0.55	1.79				cT3b,cN0,cM0	4+3	ADT + local RT

- Data are not available

Table S2. Analysis of blood plasma metabolites and PSA-DT in PCa patients before and after radiotherapy, μmol/L

	1	2	3	4	5	6
**Study ID**	**1-methyl-histidine**	**3-methyl-histidine**	**alpha aminoadipic acid**	**alpha aminobutyric acid**	**alanine**	**arginine**
HT1	5.7	0	2.7	7.3	147.3	34.1
HT2	0	2.4	3.4	10.8	198.9	19.7
HT3	5.6	0	3.4	6.5	130.1	14.3
HT4	0	0	0	2.1	56	6.6
HT6	30	5.7	5.3	14	160.6	26.1
HT7	6.7	0.9	1.4	4.5	105.1	29.3
HT8	3.7	0	4.5	3.7	160.4	19.3
HT9	3.7	0	3.6	3.3	151.2	15.9
HT10	4.4	1.3	2.2	6.3	184.2	16.1
HT11	5.1	0	0	3.5	108.1	14.2
HT12	0	1.4	0	6.9	80.7	11.6
HT13	7.2	12.7	0.8	10	120.7	19.6
HT15	5.4	5.2	0.8	6.8	94.3	14.9
HT16	1.6	3.3	1	5.8	154.4	28
HT17	1.9	0	2.1	4.6	161.5	12.8
HT18	3.6	0	1.9	2.1	120.4	12
HT21	0	0	0	5.4	78.5	19.3
HT22	0	0	5.3	7.7	193.1	6.4
HT23	7.1	0	0	2.9	119.5	5.3
HT24	3.8	0	0.9	7	135.9	22.8

Correlation with PSA DT before RT

**Pearson r**	0.07208	0.1667	0.05045	0.06993	0.3414	-0.3386
**95% confidence interval**	-0.3827 to 0.4988	-0.2979 to 0.5674	-0.4011 to 0.4823	-0.3846 to 0.4972	-0.1193 to 0.6811	-0.6794 to 0.1224
**R square**	0.005196	0.02777	0.002545	0.004891	0.1165	0.1146
**P value (two-tailed)**	0.7627	0.4825	0.8327	0.7695	0.1408	0.1443
**P value summary**	ns	ns	ns	ns	ns	ns
**Significant? (alpha = 0.05)**	No	No	No	No	No	No
**Number of XY Pairs**	20	20	20	20	20	20

Correlation with PSA DT after RT

**Pearson r**	-0.03787	-0.03828	0.2392	-0.2338	0.3246	-0.1612
**95% confidence interval**	-0.5398 to 0.4839	-0.5401 to 0.4836	-0.3113 to 0.6695	-0.6664 to 0.3165	-0.2252 to 0.7176	-0.6222 to 0.3828
**R square**	0.001434	0.001466	0.05722	0.05467	0.1054	0.02599
**P value (two-tailed)**	0.8934	0.8923	0.3906	0.4016	0.2379	0.566
**P value summary**	ns	ns	ns	ns	ns	ns
**Significant? (alpha = 0.05)**	No	No	No	No	No	No
**Number of XY Pairs**	15	15	15	15	15	15

7	8	9	10	11	12	13	14	15
**asparagine**	**aspartic acid**	**beta-aminoisobutyric acid**	**carnosine**	**citrulline**	**cystine**	**cystathionine**	**gamma-aminobutyric acid**	**glutamine**
16.5	5	0	0	8.7	0	0.4	0	246.1
23	4	0	0	7.2	2	0.5	10.6	117.8
11	4	0	0	6.6	0	0	0	163.7
5.8	5.2	4.4	0	4.2	2.4	0	0	72.3
18.2	4.5	0	0	16.6	1.3	0	5.7	203.9
8.1	3	3.1	0	7.2	0.6	0	4.5	173.4
9.4	3.6	2.8	0	4	0	0	5.2	131.7
9.7	2.2	0	0	9.9	1.9	0.5	1.7	121.3
9.6	3.7	0	0	11.9	5.3	0	13	160.7
11.8	3.5	13.4	0	5.6	0	0	0	163.4
10.3	4.3	0	0	5.3	1.4	0	9.1	212.7
11.1	5.9	0	0	10	1.8	0	3.4	177
9.3	2.5	0	0	6.8	2.4	0	0	158.8
14.3	2.4	0	0	15.8	7.7	0	6.7	202.5
10.8	5.8	0	0	8.2	0	0	0	180.6
11.3	3	8.1	0	12.6	1.1	0	0	182.8
0	5.2	1.6	5.8	1.8	0	119.3	52.5	59.8
8.7	3.9	0	1	6.4	3.6	0	0	204
2.8	7.2	0	0	3.2	6.3	0	142.4	26.1
15.4	5.8	0	0	3.8	0	1.4	75.5	194.4

-0.07989	0.3814	-0.2194	-0.0963	-0.06296	0.5107	-0.1089	0.6044	-0.5075
-0.5047 to 0.3760	-0.07371 to 0.7050	-0.6034 to 0.2472	-0.5169 to 0.3617	-0.4919 to 0.3905	0.08804 to 0.7776	-0.5261 to 0.3507	0.2209 to 0.8261	-0.7758 to -0.08367
0.006382	0.1454	0.04814	0.009273	0.003964	0.2609	0.01185	0.3654	0.2575
0.7378	0.0971	0.3527	0.6863	0.792	0.0214	0.6478	0.0048	0.0224
ns	ns	ns	ns	ns	*	ns	**	*
No	No	No	No	No	Yes	No	Yes	Yes
20	20	20	20	20	20	20	20	20

-0.05716	-0.1913	0.2346	-0.1072	0.2027	0.0857	-0.1084	-0.00657	-0.02764
-0.5533 to 0.4689	-0.6409 to 0.3559	-0.3157 to 0.6668	-0.5873 to 0.4287	-0.3456 to 0.6478	-0.4463 to 0.5729	-0.5881 to 0.4277	-0.5172 to 0.5075	-0.5325 to 0.4917
0.003267	0.03661	0.05503	0.01149	0.04107	0.007345	0.01174	0.00004316	0.0007638
0.8397	0.4945	0.4	0.7038	0.4688	0.7614	0.7007	0.9815	0.9221
ns	ns	ns	ns	ns	ns	ns	ns	ns
No	No	No	No	No	No	No	No	No
15	15	15	15	15	15	15	15	15

16	17	18	19	20	21	22	23	24	25
**glutamic acid**	**glycine**	**histidine**	**isoleucine**	**leucine**	**lysine**	**methionine**	**hydroxyproline**	**ornithine**	**phosphoetanolamine**
19.1	69.3	30.6	34.2	51.5	65.1	10.5	3	30.3	10
142.8	92.7	27.6	33.2	51.3	67.3	10.8	10.3	41.5	9.3
22.2	61.7	20.5	23.3	45.2	49.4	3.8	5.6	32.5	12.3
12.6	30.3	7.6	13.8	15.2	40.9	3.2	11.4	15.7	3.2
32.5	90.2	23.7	75.2	115.5	98.7	16.7	39	53.3	8.7
24.9	61.2	15	32.5	39	48.5	5.6	1.4	42.6	11.6
22.5	62.2	20.7	29.5	51.6	56.8	7.9	8.9	22.2	5.3
35.9	40	10.7	25.3	48.4	54.3	7.6	5.8	22.7	9.5
25.6	136.9	19.1	22.7	40.6	41.4	6	5.2	25.4	7.7
23.4	91.7	20.6	18.3	27.8	47	5.2	0	23.6	8
17.7	43.6	16.8	30.5	58.6	54.6	6.8	0.6	27.6	6.3
50.8	50.5	21.7	23.2	42.6	42.6	2	0	19.9	1.8
31	81.5	18.7	41.8	70	46.3	5.1	4.4	27	8.8
24.7	78.1	30.1	23	40.5	69	8.3	4	23.9	9.4
19.9	83.1	20.8	24	41.3	43.1	5.6	20.3	22.2	10.3
23	63.1	23.2	13.4	31.2	39.5	3.9	16.7	22.2	7.9
20	29.3	30.9	49.8	3.2	16.4	15.8		3	60.5
13.7	90.5	14.9	26.1	34.6	51.7	6.1	15.4	23.6	1.9
57.3	22.2	37.1	46.2	63.7	11.1	6.3	40.9		31.1
19.9	68.5	26.7	27.4	39.7	53.3	12.1	4.6	20.9	6.5

0.6107	-0.04078	0.4546	0.2243	0.2526	-0.3188	-0.05187	0.5585	0.1539	0.1784
0.2303 to 0.8292	-0.4748 to 0.4092	0.01496 to 0.7469	-0.2424 to 0.6067	-0.2139 to 0.6253	-0.6673 to 0.1441	-0.4834 to 0.3999	0.1396 to 0.8078	-0.3230 to 0.5685	-0.2869 to 0.5756
0.3729	0.001663	0.2066	0.0503	0.06381	0.1016	0.00269	0.3119	0.02369	0.03183
0.0042	0.8645	0.0441	0.3418	0.2826	0.1707	0.8281	0.0129	0.5293	0.4517
**	ns	*	ns	ns	ns	ns	*	ns	ns
Yes	No	Yes	No	No	No	No	Yes	No	No
20	20	20	20	20	20	20	19	19	20

0.09475	0.2699	0.01212	-0.2699	-0.1033	-0.1873	-0.2076	0.1008	-0.09838	-0.1575
-0.4389 to 0.5790	-0.2814 to 0.6872	-0.5034 to 0.5213	-0.6872 to 0.2814	-0.5847 to 0.4319	-0.6384 to 0.3596	-0.6508 to 0.3410	-0.4541 to 0.5995	-0.5814 to 0.4360	-0.6198 to 0.3860
0.008977	0.07284	0.000147	0.07284	0.01067	0.03506	0.04311	0.01017	0.009679	0.0248
0.737	0.3306	0.9658	0.3306	0.7141	0.504	0.4578	0.7316	0.7272	0.5752
ns	ns	ns	ns	ns	ns	ns	ns	ns	ns
No	No	No	No	No	No	No	No	No	No
15	15	15	15	15	15	15	14	15	15

26	27	28	29	30	31	32	33	34	35
**phenylalanine**	**proline**	**phosphoserine**	**serine**	**taurine**	**threonine**	**tryptophan**	**tyrosine**	**urea**	**valine**
29	112.3	7.2	34.5	19.1	65.7	11.4	34	3.1	105.5
88.3	112	10.3	43.1	46.8	54.8	20.4	35.4	3	91.9
45.6	90.7	8.1	24.9	18	26.3	15.3	30.3	3.4	85.6
14.5	33.7	7.7	17.6	25.3	20.5	4.4	11.3	1.1	35.2
49.4	102.3	5	45.4	24.3	67.9	36.2	48.4	3.9	189.3
24.8	76	7.3	32.9	19.5	37.5	6.8	34.5	3.9	75.3
21.6	67.8	7.7	33.2	60.9	37.4	17.2	31.6	2.1	83.9
25.1	47	6.5	20.3	22.5	23.7	8.8	27.8	2.5	89.3
31.9	87.3	6	38.9	21.8	41	10.7	26	3	84.4
19.3	64.9	6.4	28.1	18.7	35.8	2.1	25.9	2.1	55.7
30.9	71.9	8.3	32.5	14.3	38.6	0	28.2	2.1	90.4
23.5	51.7	7.7	27.9	82	23.6	12.7	18.9	3.4	77.4
23.3	42.8	7.2	40.9	28.4	32.6	15.9	22	2.7	126.1
28.3	85.6	7.9	35.3	22.6	38.7	12.2	34.5	5	84.4
22.9	73.3	8.1	29.2	21.7	29.5	13.5	25.5	1.6	84.2
22.8	50.9	9.8	29.9	21.6	25.6	9.9	23.1	2.4	66.6
8.8	29.4	13	20.8	2.7	22.8	2.1	76.8	1	2.9
27.3	89.9	7.3	37.9	19.6	47.5	0	29.5	2.1	78.5
129.8	6.6	37.5	51.3	39.9	1.9	33.4	1.9	95.8	1
27.5	97.7	6.5	39.5	14.1	48.8	17.3	32.7	2.4	81.3

0.8282	-0.3019	0.7685	0.592	0.5301	-0.3153	0.5605	-0.3796	0.7796	-0.2654
0.6087 to 0.9299	-0.6567 to 0.1625	0.4939 to 0.9037	0.2025 to 0.8198	0.1144 to 0.7879	-0.6651 to 0.1479	0.1568 to 0.8037	0.7039 to 0.0757	0.5146 to 0.9087	0.6336 to 0.2008
0.6859	0.09112	0.5906	0.3505	0.281	0.09944	0.3142	0.1441	0.6078	0.07043
< 0.0001	0.1958	< 0.0001	0.006	0.0162	0.1756	0.0101	0.0988	< 0.0001	0.2581
****	ns	****	**	*	ns	*	ns	****	ns
Yes	No	Yes	Yes	Yes	No	Yes	No	Yes	No
20	20	20	20	20	20	20	20	20	20

-0.06295	-0.07165	0.08505	0.1591	0.3151	-0.1183	0.07318	-0.1484	-0.02575	-0.09277
-0.5573 to 0.4644	-0.5633 to 0.4575	-0.4468 to 0.5725	-0.3846 to 0.6209	-0.2352 to 0.7125	-0.5946 to 0.4195	-0.4563 to 0.5644	-0.6141 to 0.3939	0.5311 to 0.493	0.5777 to 0.4405
0.003963	0.005133	0.007233	0.02533	0.09931	0.01399	0.005355	0.02202	0.0006632	0.008606
0.8236	0.7997	0.7631	0.571	0.2526	0.6746	0.7955	0.5977	0.9274	0.7423
ns	ns	ns	ns	ns	ns	ns	ns	ns	ns
No	No	No	No	No	No	No	No	No	No
15	15	15	15	15	15	15	15	15	15

Table S3. Clinical characteristics of the patients included in the *MYC* gene expression analysis depicted on Figure 8E.

Parameter	Number of patients	%
		In total: 74 patients, treated in 2000-2011
Gleason score<br>≤6/7/8/9/missing	41/23/5/4/1	55.4/31.1/6.8/5.4/1.4
T stage<br>1/2/3/4	44/21/8/1	59.4/28.4/10.8/1.4
D'Amico risk score<br>low/intermediate/high/missing	24/26/23/1	32.4/35.1/31.1/1.4
Hormone therapy<br>yes/no	40/34	54.1/45.9
Radiotherapy<br>external beam/low dose rate brachy therapy	60/14	81.1/18.9
**Parameter**	**Median**	**Range**
Age (years)	71.2	55.5-85.0
Dose (Gy)	76.0	70.2-145.0
Tumour volume (ml)	34.0	16.0-121.0 (missing: 11)
PSA initial (ng/ml)	7.3	0.39-62.4
Follow-up time all patients (months)	116.3	25.7-187.4

Table S4. Characteristics of PCa patients (n = 14). Primary prostate cancer and adjacent normal tissues (benign hyperplasia, BPH) from these patients were used for primary tissue cultures and radiobiological colony forming assays.

ID	Gleason score	TNM staging	PSA (ng/ml)	Age
BM_PC_11 RR	5+5	pT3ay pN0	38,0 ng/ml	64
BM_PC_12 RR	4+3	pT2c pN0	8,87 ng/ml	76
BM_PC_14* RR	3+3	pT2c pN0	7,85 ng/ml	68
BM_PC_16* RR	3+4	pT3a pN0	22,42 ng/ml	63
BM_PC_19 RS	4+3	cT1c	7,17 ng/ml	73
BM_PC_20 RS	4+3	pT3b pN0	27,25 ng/ml	65
BM_PC_21 RR	3+3	pT3b pN1	8,11 ng/ml	73
BM_PC_22	4+3	pT3b,pN0	8,77 ng/ml	68
BM_PC_23 RS	5+4	pT3b pN1	22,11 ng/ml	65
BM_PC_25 RS	3+4	pT2c pN0	6,4 ng/ml	62
BM_PC_26 RS	4+3	pT3b pN0	8,21 ng/ml	76
BM_PC_28 RS	3+3	pT3a, pN0	15,3 ng/ml	66
BM_PC_31	4+5	pT3b, pN0	5,14 ng/ml	68
BM_PC_42	3+4	pT2c pN0	10,38 ng/ml	69

RR (green)	specimens used for the experiments depicted on Figure 2 g and characterized as radioresistant
RS (green)	specimens used for the experiments depicted on Figure 2 g and characterized as radiosensitive
(pink)	specimens used for the experiments depicted on Figure 3 e
	* specimens used for both types of experiments

Table S5. Nucleotide sequences of siRNAs, primers used for qPCR and antibodies used for western blotting and fluorescence microscopy

**siRNA**	**Target sequence**
Scr #1	5'-GGCUAAAGGAAACGAAAGA -3'
Scr #2	5'-UGCGCUAGGCCUCGGUUGC -3'
Scr #3	5'-GCAGCUAUAUGAAUGUUGU -3'
c-Myc #1	5'-GGAACGAGCUAAAACGGAG-3'
c-Myc #2	5'-GGAGGAGAACUUCUACCAG-3'
c-Myc #3	5'-CGACGAGACCUUCAUCAAA-3'
GLS #1	5'-GCAGUUCGAAAUACAUUGA-3'
GLS #2	5'-GGGUCUGUUACCUAGCUUG -3'
GLS #3	5'-GGACAAGAGAAAAUACCUG -3'
ATG5#1	5'-GGAUGAGAUAACUGAAAGG -3'
ATG5#2	5'-GGCAACCUGACCAGAAACA -3'
**Oligonucleotide primers**	**Nucleotide sequences**
c-Myc	Forward: 5'-CTCCGTCCTCGGATTCTCTGC-3'<br>Reverse: 5'-CTCCAGCAGAAGGTGATCCAG-3'
ACTB	Forward: 5'-ATGGAGTCCTGTGGCATCCA-3'<br>Reverse: 5'-AGTACTTGCGCTCAGGAGGA-3'
ATF3	Forward: 5'- GTGAATGCTGAACTGAAGGC -3'<br>Reverse: 5'- CCATACCACGACTGCTTAGC -3'
ALKBH1	Forward: 5'- AGAAGCGACTAAACGGAGACC -3'<br>Reverse: 5'- GGGAAAGGTGTGTAATGATCTGC -3'
ALKBH3	Forward: 5'- GCTTGTGTCGCTCCTTCCAT -3'<br>Reverse: 5'- ACACGCACATTTGAGATGAGAA -3'
ATF4	Forward: 5'- GGTCAGTCCCTCCAACAACAG -3'<br>Reverse: 5'- GACTAGGGGGGCAAAGAGATCAC -3'
GADD34	Forward: 5'- GAGACAGAGGAAGAGGAAGCT -3'<br>Reverse: 5'- GGAAATGGACAGTGACCTTCTC -3'
GLS1	Forward: 5'- GAGGCATTCTACTGGAGATACC-3'<br>Reverse: 5'- GCTCCAGCATTTACCATAGG-3'
IRE1	Forward: 5'- CAGAATTGGTGCAGGCATCCC-3'<br>Reverse: 5'- GGTGTCGTACATGGTGATGGTG-3'

**Antibodies / application**	**Manufacturer ID**
ACTB / Western blotting	Cell Signaling Technology, #4967
ALDH1A1 / Immunofluorencence microscopy	Santa Cruz Biotechnology, (H-4), #sc-374076
ATG5 / Western blotting	Cell Signaling Technology, #12994
ATM / Western blotting	Cell Signaling Technology, #2873
ATR / Western blotting	Cell Signaling Technology, #13934
pATR / Western blotting	Cell Signaling Technology, #2853
Chk1 / Western blotting	Cell Signaling Technology, #2360
pChk1 S296 / Western blotting	Cell Signaling Technology, #2349
GAPDH / Western blotting	Santa Cruz Biotechnology, #sc-25778
GLS / Western blotting	Cell Signaling Technology, #88964
γH2A.X / Western blotting, Immunofluorencence microscopy	Merck KGaA, # 05-636
H3 / Western blotting	Cell Signaling Technology, #2650
H3K27me3 / Western blotting	Cell Signaling Technology, #9733
H3K36me3 / Western blotting	Cell Signaling Technology, #4909
H3K9me3 / Western blotting	Cell Signaling Technology, #13969
LC3B / Western blotting, Immunofluorencence microscopy	Cell Signaling Technology, #3868
c-Myc / Western blotting	Cell signaling Technology, #9402
PARP / Western blotting	Cell Signaling Technology, #9532
cleaved-PARP / Western blotting	Cell Signaling Technology, #5625
Tubulin / Western blotting	Cell Signaling Technology, #3873

Table S6. Targeted metabolomics of amino acids and biogenic amines

Concentration [**µM**]

Cell Lines/Irradiation	Total Protein Mass	Ala	Arg	Asn	Asp	Cit	Gln
DU145 p9, line A	56	133	2.9	15.2	33.6	< LOD	< LOD
DU145 p9, line A	56	144	2.54	15.8	18.7	< LOD	< LOD
DU145 p9, line B	44	102	4.36	11.8	34.2	< LOD	8.37
DU145 p9, line B	44	105	2.99	12.7	32.8	< LOD	3.49
DU145 RR p9, line A, 21x4Gy, Irradiated 25.01.16	35	77.6	1.52	16.4	18.4	< LOD	27.4
DU145 RR p9, line A, 21x4Gy, Irradiated 25.01.17	35	76.4	2.6	16.9	24.5	< LOD	36.9
DU145 RR p9, line B, 21x4Gy, Irradiated 25.01.18	48	77.6	4.72	14.3	25.1	< LOD	35.1
DU145 RR p9, line B, 21x4Gy, Irradiated 25.01.19	48	76.7	3.59	15.1	21.7	< LOD	34.3
LNCaP p9, line A	32	102	13.9	43.1	22	< LOD	27.8
LNCaP p9, line A	32	113	18.5	47.3	18.3	< LOD	30.5
LNCaP p9, line B	29	87.6	12.6	37.4	19.4	< LOD	27.4
LNCaP p9, line B	29	108	23.8	48.9	27.5	< LOD	32.7
LNCaP RR p9, line A, 21x4Gy, Irradiated 25.01.16	50	126	16.1	39.9	5.59	< LOD	< LOD
LNCaP RR p9, line A, 21x4Gy, Irradiated 25.01.17	50	129	17.5	44.8	3.29	< LOD	2.68
LNCaP RR p9, line B, 21x4Gy, Irradiated 25.01.18	44	102	17.5	42.1	11	< LOD	23.2
LNCaP RR p9, line B, 21x4Gy, Irradiated 25.01.19	44	89.8	13.6	37.6	9.68	< LOD	20
PC3 p8, line A	26	27.6	4.93	5.54	29.9	< LOD	64.4
PC3 p8, line A	26	34.7	9.29	7.04	31.5	< LOD	78.7
PC3 p8, line B	33	32.2	6.61	6.97	25.5	< LOD	65.8
PC3 p8, line B	33	28.9	2.76	5.29	27.5	< LOD	53.7
PC3 RR p8, line A, 14x4Gy, Irradiated 27.01.16	19	21.9	2.54	4.41	27.6	< LOD	40.3
PC3 RR p8, line A, 14x4Gy, Irradiated 27.01.17	19	21.1	2	4.39	24.6	< LOD	45.1
PC3 RR p8, line B, 14x4Gy, Irradiated 27.01.18	18	23.6	4.04	4.73	33.1	< LOD	54.6
PC3 RR p8, line B, 14x4Gy, Irradiated 27.01.19	18	24.8	1.6	3.79	21.8	< LOD	48.9
DU145 p9, line A + 24h irradiated	52	72.9	1.27	7.24	< LOD	< LOD	< LOD
DU145 p9, line A + 24h irradiated	52	100	1.93	10.3	< LOD	< LOD	< LOD
DU145 RR p9, line A, 21x4Gy, Irradiated 25.01.16 + 24h irradiated	77	92.3	0.888	13.6	8.32	< LOD	< LOD
DU145 RR p9, line A, 21x4Gy, Irradiated 25.01.16 + 24h irradiated	77	98.1	1.05	13.9	12.2	< LOD	< LOD
Medium control		< LOD	< LOD	< LOD	< LOD	< LOD	< LOD
Medium control		< LOD	< LOD	< LOD	< LOD	< LOD	< LOD

Concentration [**pmol/10^6 cells**]

Cell Lines/Irradiation	Total Protein Mass	Ala	Arg	Asn	Asp	Cit	Gln
DU145 p9, line A	56	23750000	517857.1429	2714285.714	6000000	< LOD	< LOD
DU145 p9, line A	56	25714285.71	453571.4286	2821428.571	3339285.714	< LOD	< LOD
DU145 p9, line B	44	23181818.18	990909.0909	2681818.182	7772727.273	< LOD	1902272.727
DU145 p9, line B	44	23863636.36	679545.4545	2886363.636	7454545.455	< LOD	793181.8182
DU145 RR p9, line A, 21x4Gy, Irradiated 25.01.16	35	22171428.57	434285.7143	4685714.286	5257142.857	< LOD	7828571.429
DU145 RR p9, line A, 21x4Gy, Irradiated 25.01.17	35	21828571.43	742857.1429	4828571.429	7000000	< LOD	10542857.14
DU145 RR p9, line B, 21x4Gy, Irradiated 25.01.18	48	16166666.67	316666.6667	3416666.667	3833333.333	< LOD	5708333.333
DU145 RR p9, line B, 21x4Gy, Irradiated 25.01.19	48	15916666.67	541666.6667	3520833.333	5104166.667	< LOD	7687500
LNCaP p9, line A	32	31875000	4343750	13468750	6875000	< LOD	8687500
LNCaP p9, line A	32	35312500	5781250	14781250	5718750	< LOD	9531250
LNCaP p9, line B	29	30206896.55	4344827.586	12896551.72	6689655.172	< LOD	9448275.862
LNCaP p9, line B	29	37241379.31	8206896.552	16862068.97	9482758.621	< LOD	11275862.07
LNCaP RR p9, line A, 21x4Gy, Irradiated 25.01.16	50	25200000	3220000	7980000	1118000	< LOD	< LOD
LNCaP RR p9, line A, 21x4Gy, Irradiated 25.01.17	50	25800000	3500000	8960000	658000	< LOD	536000
LNCaP RR p9, line B, 21x4Gy, Irradiated 25.01.18	44	23181818.18	3977272.727	9568181.818	2500000	< LOD	5272727.273
LNCaP RR p9, line B, 21x4Gy, Irradiated 25.01.19	44	20409090.91	3090909.091	8545454.545	2200000	< LOD	4545454.545
PC3 p8, line A	26	10615384.62	1896153.846	2130769.231	11500000	< LOD	24769230.77
PC3 p8, line A	26	13346153.85	3573076.923	2707692.308	12115384.62	< LOD	30269230.77
PC3 p8, line B	33	9757575.758	2003030.303	2112121.212	7727272.727	< LOD	19939393.94
PC3 p8, line B	33	8757575.758	836363.6364	1603030.303	8333333.333	< LOD	16272727.27
PC3 RR p8, line A, 14x4Gy, Irradiated 27.01.16	19	11526315.79	1336842.105	2321052.632	14526315.79	< LOD	21210526.32
PC3 RR p8, line A, 14x4Gy, Irradiated 27.01.17	19	11105263.16	1052631.579	2310526.316	12947368.42	< LOD	23736842.11
PC3 RR p8, line B, 14x4Gy, Irradiated 27.01.18	18	13111111.11	2244444.444	2627777.778	18388888.89	< LOD	30333333.33
PC3 RR p8, line B, 14x4Gy, Irradiated 27.01.19	18	13777777.78	888888.8889	2105555.556	12111111.11	< LOD	27166666.67
DU145 p9, line A + 24h irradiated	52	14019230.77	244230.7692	1392307.692	< LOD	< LOD	< LOD
DU145 p9, line A + 24h irradiated	52	19230769.23	371153.8462	1980769.231	< LOD	< LOD	< LOD
DU145 RR p9, line A, 21x4Gy, Irradiated 25.01.16 + 24h irradiated	77	11987012.99	115324.6753	1766233.766	1080519.481	< LOD	< LOD
DU145 RR p9, line A, 21x4Gy, Irradiated 25.01.16 + 24h irradiated	77	12740259.74	136363.6364	1805194.805	1584415.584	< LOD	< LOD

Cell Lines/Irradiation	p-values (t-test)	Ala	Arg	Asn	Asp	Cit	Gln
DU145 vs DU145 RR		0.030162485	0.352027018	0.012079201	0.50939231		0.012481778
LNCaP vs LNCaP RR		0.002491473	0.054468031	0.00088859	0.000894671		0.006314457
PC3 vs PC3 RR		0.183689218	0.318175256	0.448306837	0.042075717		0.469804156
DU145 vs DU145 + 4Gy, 24 h		0.013950174	0.127818838	0.004912586			
DU145 RR vs DU145 RR + 4Gy, 24 h		0.06218887	0.047940296	0.014248204	0.015949674		

Glu	Gly	His	Ile	Leu	Lys	Met	Orn	Phe	Pro	Ser	Thr
216	123	6.58	18.9	17.9	2.2	8.53	< LOD	13.7	30.1	92.8	129
202	151	7.86	23.9	29.5	1.68	11.3	< LOD	15.9	32.8	108	139
254	111	7.8	26.3	41.1	6.68	11.3	< LOD	15.8	26.3	95.3	102
266	104	6.64	21.2	17.5	2.05	8.48	< LOD	13.8	25.5	90.4	103
357	127	5.12	19.1	20.7	< LOD	5.06	< LOD	13.3	43.2	53.2	85.5
384	145	6.62	23.5	33	2.86	5.82	< LOD	15.5	48.7	60.6	85
346	144	7.78	28.4	19	7.67	7.01	< LOD	17.5	44.9	64.5	86.7
357	141	6.92	24.7	15.8	5.13	6.09	< LOD	15.1	45.8	62.8	86
111	131	0.759	6.47	10.9	< LOD	0.207	5.98	1.45	25.4	52.3	19.2
97.2	165	1.67	7.6	12.2	1.63	0.162	10.4	2.34	30.5	62.4	21.3
104	100	< LOD	5.05	9.81	< LOD	< LOD	4.82	1.24	18.3	46.2	17.3
125	129	2.32	7.97	21.3	4.36	0.567	13.5	3.26	27.1	57.8	22.1
73.3	221	0.833	5.31	10.6	< LOD	< LOD	7.13	1.53	37.9	39.9	20.5
82.4	244	1.1	5.66	6.36	< LOD	< LOD	10.3	1.82	43.6	45.1	21.5
115	151	1.05	5.9	7.03	< LOD	0.22	6.33	1.92	31.9	50.1	19.7
110	141	< LOD	4.72	5.26	< LOD	< LOD	4.16	1.44	28.9	44.2	17.2
130	51.9	5.15	17.3	15.1	6.8	7.38	< LOD	11.6	9.95	33.5	43.8
138	64.1	7.88	24.2	30.8	17.3	8.33	< LOD	15.6	11.6	42.5	54.9
135	55.7	6.01	17.9	25.5	10.7	7.8	< LOD	12.7	9.73	32.5	50.8
129	53.2	4.69	17.4	15.9	1.22	7.51	< LOD	10.4	10.1	31.5	47
153	31	3.59	14.4	12.4	1.77	4.3	< LOD	8.21	5.52	24	31.9
136	33.1	3.51	13.6	10.1	0.97	4.48	< LOD	8.05	5.74	25.5	33.3
146	35	4.68	16.7	27.4	5.32	5.16	< LOD	10.2	6.37	28.2	37.8
162	35.3	3.55	12.5	18.6	< LOD	4.39	< LOD	8.42	6.26	27.1	35.1
15.4	87.3	4.54	13.7	19.2	< LOD	6.17	< LOD	11.2	20	62.9	83.5
37.5	110	6.62	19.7	13.7	< LOD	7.97	< LOD	13.2	24.5	72.8	108
253	131	3.32	12.8	11.5	< LOD	2.84	< LOD	10.4	49.4	33.1	83.3
287	156	4.49	17.9	16.6	< LOD	3.32	< LOD	12	55.6	42.2	93.8
< LOD	< LOD	< LOD	< LOD	< LOD	< LOD	< LOD	< LOD	< LOD	< LOD	< LOD	< LOD
< LOD	< LOD	< LOD	< LOD	2.8	< LOD	< LOD	< LOD	< LOD	< LOD	< LOD	< LOD

Glu	Gly	His	Ile	Leu	Lys	Met	Orn	Phe	Pro	Ser	Thr
38571428.57	21964285.71	1175000	3375000	3196428.571	392857.1429	1523214.286	< LOD	2446428.571	5375000	16571428.57	23035714.29
36071428.57	26964285.71	1403571.429	4267857.143	5267857.143	300000	2017857.143	< LOD	2839285.714	5857142.857	19285714.29	24821428.57
57727272.73	25227272.73	1772727.273	5977272.727	9340909.091	1518181.818	2568181.818	< LOD	3590909.091	5977272.727	21659090.91	23181818.18
60454545.45	23636363.64	1509090.909	4818181.818	3977272.727	465909.0909	1927272.727	< LOD	3136363.636	5795454.545	20545454.55	23409090.91
102000000	36285714.29	1462857.143	5457142.857	5914285.714		1445714.286	< LOD	3800000	12342857.14	15200000	24428571.43
109714285.7	41428571.43	1891428.571	6714285.714	9428571.429	817142.8571	1662857.143	< LOD	4428571.429	13914285.71	17314285.71	24285714.29
74375000	26458333.33	1066666.667	3979166.667	4312500		1054166.667	< LOD	2770833.333	9000000	11083333.33	17812500
80000000	30208333.33	1379166.667	4895833.333	6875000	595833.3333	1212500	< LOD	3229166.667	10145833.33	12625000	17708333.33
34687500	40937500	237187.5	2021875	3406250	< LOD	64687.5	1868750	453125	7937500	16343750	6000000
30375000	51562500	521875	2375000	3812500	509375	50625	3250000	731250	9531250	19500000	6656250
35862068.97	34482758.62	< LOD	1741379.31	3382758.621	< LOD	< LOD	1662068.966	427586.2069	6310344.828	15931034.48	5965517.241
43103448.28	44482758.62	800000	2748275.862	7344827.586	1503448.276	195517.2414	4655172.414	1124137.931	9344827.586	19931034.48	7620689.655
14660000	44200000	166600	1062000	2120000	< LOD	< LOD	1426000	306000	7580000	7980000	4100000
16480000	48800000	220000	1132000	1272000	< LOD	< LOD	2060000	364000	8720000	9020000	4300000
26136363.64	34318181.82	238636.3636	1340909.091	1597727.273	< LOD	50000	1438636.364	436363.6364	7250000	11386363.64	4477272.727
25000000	32045454.55	< LOD	1072727.273	1195454.545	< LOD	< LOD	945454.5455	327272.7273	6568181.818	10045454.55	3909090.909
50000000	19961538.46	1980769.231	6653846.154	5807692.308	2615384.615	2838461.538	< LOD	4461538.462	3826923.077	12884615.38	16846153.85
53076923.08	24653846.15	3030769.231	9307692.308	11846153.85	6653846.154	3203846.154	< LOD	6000000	4461538.462	16346153.85	21115384.62
40909090.91	16878787.88	1821212.121	5424242.424	7727272.727	3242424.242	2363636.364	< LOD	3848484.848	2948484.848	9848484.848	15393939.39
39090909.09	16121212.12	1421212.121	5272727.273	4818181.818	369696.9697	2275757.576	< LOD	3151515.152	3060606.061	9545454.545	14242424.24
80526315.79	16315789.47	1889473.684	7578947.368	6526315.789	931578.9474	2263157.895	< LOD	4321052.632	2905263.158	12631578.95	16789473.68
71578947.37	17421052.63	1847368.421	7157894.737	5315789.474	510526.3158	2357894.737	< LOD	4236842.105	3021052.632	13421052.63	17526315.79
81111111.11	19444444.44	2600000	9277777.778	15222222.22	2955555.556	2866666.667	< LOD	5666666.667	3538888.889	15666666.67	21000000
90000000	19611111.11	1972222.222	6944444.444	10333333.33	< LOD	2438888.889	< LOD	4677777.778	3477777.778	15055555.56	19500000
2961538.462	16788461.54	873076.9231	2634615.385	3692307.692	< LOD	1186538.462	< LOD	2153846.154	3846153.846	12096153.85	16057692.31
7211538.462	21153846.15	1273076.923	3788461.538	2634615.385	< LOD	1532692.308	< LOD	2538461.538	4711538.462	14000000	20769230.77
32857142.86	17012987.01	431168.8312	1662337.662	1493506.494	< LOD	368831.1688	< LOD	1350649.351	6415584.416	4298701.299	10818181.82
37272727.27	20259740.26	583116.8831	2324675.325	2155844.156	< LOD	431168.8312	< LOD	1558441.558	7220779.221	5480519.481	12181818.18

Glu	Gly	His	Ile	Leu	Lys	Met	Orn	Phe	Pro	Ser	Thr
0.006454703	0.038876667	0.945250849	0.44053649	0.519827163	0.935554753	0.039130357		0.247536515	0.002328088	0.021075263	0.237969921
0.007800999	0.591865919	0.130364886	0.003329638	0.024058791			0.105786587	0.095142295	0.421781935	0.00060389	0.001135342
0.000457772	0.586036624	0.972674965	0.35516379	0.532574725	0.339661096	0.485857978		0.620326914	0.417038867	0.285071358	0.350059228
0.01082608	0.058761109	0.153164989	0.191155675	0.333294595		0.128421243		0.159630171	0.011127705	0.020615729	0.0295318
0.011672744	0.041583889	0.022030731	0.020717233	0.041221745		0.0093226		0.017833889	0.053229272	0.012006577	0.029637855

Trp	Tyr	Val	ADMA	Ac-Orn	Carnosine	Creatinine	DOPA	Dopamine	Histamine	Kynurenine	Met-SO
1.73	14.1	20.8	< LOD	< LOD	< LOD	< LOD	< LOD	< LOD	< LOD	< LOD	< LOD
2.6	15.7	23.6	< LOD	< LOD	< LOD	< LOD	< LOD	< LOD	< LOD	< LOD	0.821
2.34	15.7	24.6	< LOD	< LOD	< LOD	< LOD	< LOD	< LOD	< LOD	< LOD	0.549
1.94	14	20.5	< LOD	< LOD	< LOD	< LOD	< LOD	< LOD	< LOD	< LOD	< LOD
1.59	12.3	18.4	< LOD	< LOD	< LOD	< LOD	< LOD	< LOD	< LOD	< LOD	< LOD
2.45	15.6	23.4	< LOD	< LOD	< LOD	< LOD	< LOD	< LOD	< LOD	< LOD	< LOD
2.75	16.7	26.5	< LOD	< LOD	< LOD	< LOD	< LOD	< LOD	0.028	< LOD	0.746
2.29	15.8	23	< LOD	< LOD	< LOD	< LOD	< LOD	< LOD	< LOD	< LOD	0.565
< LOD	1.32	< LOD	< LOD	< LOD	< LOD	< LOD	< LOD	< LOD	< LOD	< LOD	0.904
< LOD	2.39	1.87	< LOD	< LOD	< LOD	2.09	< LOD	< LOD	< LOD	< LOD	1.14
< LOD	1.07	< LOD	< LOD	< LOD	< LOD	< LOD	< LOD	< LOD	< LOD	< LOD	0.643
< LOD	3.27	3.43	< LOD	< LOD	< LOD	< LOD	< LOD	< LOD	< LOD	< LOD	0.889
< LOD	1.6	< LOD	< LOD	< LOD	< LOD	< LOD	< LOD	< LOD	< LOD	< LOD	0.871
< LOD	1.55	< LOD	< LOD	< LOD	< LOD	2.13	< LOD	< LOD	< LOD	< LOD	1.54
< LOD	1.92	0.842	< LOD	< LOD	< LOD	< LOD	< LOD	< LOD	< LOD	< LOD	0.666
< LOD	1.4	< LOD	< LOD	< LOD	< LOD	< LOD	< LOD	< LOD	< LOD	< LOD	0.988
1.69	11.4	17.6	< LOD	< LOD	< LOD	< LOD	< LOD	< LOD	< LOD	< LOD	< LOD
2.52	15.5	26	< LOD	< LOD	< LOD	< LOD	< LOD	< LOD	< LOD	< LOD	0.376
1.74	13.1	20.2	< LOD	< LOD	< LOD	< LOD	< LOD	< LOD	< LOD	< LOD	< LOD
1.21	10.7	15	< LOD	< LOD	< LOD	< LOD	< LOD	< LOD	< LOD	< LOD	< LOD
1.06	8.59	11.6	< LOD	< LOD	< LOD	< LOD	< LOD	< LOD	< LOD	< LOD	< LOD
0.869	8.55	12.5	< LOD	< LOD	< LOD	< LOD	< LOD	< LOD	< LOD	< LOD	< LOD
1.47	10.3	16	< LOD	< LOD	< LOD	< LOD	< LOD	< LOD	< LOD	< LOD	< LOD
0.856	8.08	12.3	< LOD	< LOD	< LOD	< LOD	< LOD	< LOD	< LOD	< LOD	< LOD
1.28	10.3	14.3	< LOD	< LOD	< LOD	< LOD	< LOD	< LOD	< LOD	< LOD	< LOD
1.61	13.5	19	< LOD	< LOD	< LOD	< LOD	< LOD	< LOD	< LOD	< LOD	0.584
1.23	9.62	13.6	< LOD	< LOD	< LOD	< LOD	< LOD	< LOD	< LOD	< LOD	< LOD
1.46	12.3	16.3	< LOD	< LOD	< LOD	< LOD	< LOD	< LOD	< LOD	< LOD	< LOD
< LOD	< LOD	< LOD	< LOD	< LOD	< LOD	< LOD	< LOD	< LOD	< LOD	< LOD	< LOD
< LOD	< LOD	< LOD	< LOD	< LOD	< LOD	< LOD	< LOD	< LOD	< LOD	< LOD	< LOD

Trp	Tyr	Val	ADMA	Ac-Orn	Carnosine	Creatinine	DOPA	Dopamine	Histamine	Kynurenine	Met-SO
308928.5714	2517857.143	3714285.714	< LOD	< LOD	< LOD	< LOD	< LOD	< LOD	< LOD	< LOD	< LOD
464285.7143	2803571.429	4214285.714	< LOD	< LOD	< LOD	< LOD	< LOD	< LOD	< LOD	< LOD	146607.1429
531818.1818	3568181.818	5590909.091	< LOD	< LOD	< LOD	< LOD	< LOD	< LOD	< LOD	< LOD	124772.7273
440909.0909	3181818.182	4659090.909	< LOD	< LOD	< LOD	< LOD	< LOD	< LOD	< LOD	< LOD	< LOD
454285.7143	3514285.714	5257142.857	< LOD	< LOD	< LOD	< LOD	< LOD	< LOD	< LOD	< LOD	< LOD
700000	4457142.857	6685714.286	< LOD	< LOD	< LOD	< LOD	< LOD	< LOD	< LOD	< LOD	< LOD
331250	2562500	3833333.333	< LOD	< LOD	< LOD	< LOD	< LOD	< LOD	< LOD	< LOD	< LOD
510416.6667	3250000	4875000	< LOD	< LOD	< LOD	< LOD	< LOD	< LOD	< LOD	< LOD	< LOD
< LOD	412500	< LOD	< LOD	< LOD	< LOD	< LOD	< LOD	< LOD	< LOD	< LOD	282500
< LOD	746875	584375	< LOD	< LOD	< LOD	653125	< LOD	< LOD	< LOD	< LOD	356250
< LOD	368965.5172	< LOD	< LOD	< LOD	< LOD	< LOD	< LOD	< LOD	< LOD	< LOD	221724.1379
< LOD	1127586.207	1182758.621	< LOD	< LOD	< LOD	< LOD	< LOD	< LOD	< LOD	< LOD	306551.7241
< LOD	320000	< LOD	< LOD	< LOD	< LOD	< LOD	< LOD	< LOD	< LOD	< LOD	174200
< LOD	310000	< LOD	< LOD	< LOD	< LOD	426000	< LOD	< LOD	< LOD	< LOD	308000
< LOD	436363.6364	191363.6364	< LOD	< LOD	< LOD	< LOD	< LOD	< LOD	< LOD	< LOD	151363.6364
< LOD	318181.8182	< LOD	< LOD	< LOD	< LOD	< LOD	< LOD	< LOD	< LOD	< LOD	224545.4545
650000	4384615.385	6769230.769	< LOD	< LOD	< LOD	< LOD	< LOD	< LOD	< LOD	< LOD	< LOD
969230.7692	5961538.462	10000000	< LOD	< LOD	< LOD	< LOD	< LOD	< LOD	< LOD	< LOD	144615.3846
527272.7273	3969696.97	6121212.121	< LOD	< LOD	< LOD	< LOD	< LOD	< LOD	< LOD	< LOD	< LOD
366666.6667	3242424.242	4545454.545	< LOD	< LOD	< LOD	< LOD	< LOD	< LOD	< LOD	< LOD	< LOD
557894.7368	4521052.632	6105263.158	< LOD	< LOD	< LOD	< LOD	< LOD	< LOD	< LOD	< LOD	< LOD
457368.4211	4500000	6578947.368	< LOD	< LOD	< LOD	< LOD	< LOD	< LOD	< LOD	< LOD	< LOD
816666.6667	5722222.222	8888888.889	< LOD	< LOD	< LOD	< LOD	< LOD	< LOD	< LOD	< LOD	< LOD
475555.5556	4488888.889	6833333.333	< LOD	< LOD	< LOD	< LOD	< LOD	< LOD	< LOD	< LOD	< LOD
246153.8462	1980769.231	2750000	< LOD	< LOD	< LOD	< LOD	< LOD	< LOD	< LOD	< LOD	< LOD
309615.3846	2596153.846	3653846.154	< LOD	< LOD	< LOD	< LOD	< LOD	< LOD	< LOD	< LOD	112307.6923
159740.2597	1249350.649	1766233.766	< LOD	< LOD	< LOD	< LOD	< LOD	< LOD	< LOD	< LOD	< LOD
189610.3896	1597402.597	2116883.117	< LOD	< LOD	< LOD	< LOD	< LOD	< LOD	< LOD	< LOD	< LOD

Trp	Tyr	Val	ADMA	Ac-Orn	Carnosine	Creatinine	DOPA	Dopamine	Histamine	Kynurenine	Met-SO
0.512573596	0.381930868	0.418710509									
	0.125523892										0.133736439
0.746891133	0.543826715	0.858171886									
0.094483329	0.135476741	0.111185876									
0.048389917	0.027713469	0.022500939									

Nitro-Tyr	PEA	Putrescine	SDMA	Serotonin	Spermidine	Spermine	Taurine	alpha-AAA	c4-OH-Pro	t4-OH-Pro
< LOD	< LOD	4.11	< LOD	< LOD	2.83	0.767	63	< LOD	< LOD	1.75
< LOD	< LOD	4.99	< LOD	< LOD	4.34	1.37	68.1	< LOD	< LOD	2.04
< LOD	< LOD	3.79	< LOD	< LOD	7.64	2.14	56.3	< LOD	< LOD	1.67
< LOD	< LOD	3.77	< LOD	< LOD	5.24	1.9	58.8	< LOD	< LOD	1.56
< LOD	< LOD	1.26	< LOD	< LOD	5.44	3.54	76	< LOD	< LOD	1.41
< LOD	< LOD	1.25	< LOD	< LOD	5.37	4.13	75.3	0.443	< LOD	1.5
< LOD	< LOD	1.18	< LOD	< LOD	5.52	3.94	74.4	< LOD	< LOD	1.52
< LOD	< LOD	1.29	< LOD	< LOD	6.59	5.93	71.3	< LOD	< LOD	1.42
< LOD	< LOD	2.74	< LOD	< LOD	4.37	2.98	8.24	< LOD	< LOD	15.4
< LOD	< LOD	2.59	< LOD	< LOD	4.2	2.56	8.76	< LOD	< LOD	17
< LOD	< LOD	2.74	< LOD	< LOD	5.97	2.81	7.81	< LOD	< LOD	12.7
< LOD	< LOD	2.76	< LOD	< LOD	5.38	2.1	8.7	< LOD	< LOD	16.2
< LOD	< LOD	1.5	< LOD	< LOD	2.64	3.71	10.9	< LOD	< LOD	16.4
< LOD	< LOD	1.93	< LOD	< LOD	1.88	2.31	13.1	< LOD	< LOD	18.3
< LOD	< LOD	2.01	< LOD	< LOD	5.26	5	10.9	< LOD	< LOD	16.2
< LOD	< LOD	1.56	< LOD	< LOD	3.43	3.06	10.3	< LOD	< LOD	14.2
< LOD	< LOD	4.97	< LOD	< LOD	4.47	1.9	24.4	< LOD	< LOD	0.566
< LOD	< LOD	5.65	< LOD	< LOD	5.8	2.54	27.8	< LOD	< LOD	0.676
< LOD	< LOD	5.4	< LOD	< LOD	5.62	2.08	27.2	< LOD	< LOD	0.624
< LOD	< LOD	5.51	< LOD	< LOD	4.88	1.44	27.5	< LOD	< LOD	0.577
< LOD	< LOD	2.74	< LOD	< LOD	3.92	1.86	25.7	< LOD	< LOD	0.37
< LOD	< LOD	3.09	< LOD	< LOD	4.48	1.96	26.9	< LOD	< LOD	0.454
< LOD	< LOD	3.06	< LOD	< LOD	4.67	1.76	29.6	< LOD	< LOD	0.492
< LOD	< LOD	2.99	< LOD	< LOD	4.33	1.6	30.8	< LOD	< LOD	0.508
< LOD	< LOD	2.48	< LOD	< LOD	1.61	0.511	24.4	< LOD	< LOD	1.25
< LOD	< LOD	2.46	< LOD	< LOD	1.67	0.463	31.4	< LOD	< LOD	1.33
< LOD	< LOD	0.853	< LOD	< LOD	0.927	0.68	38.5	< LOD	< LOD	1.37
< LOD	< LOD	0.912	< LOD	< LOD	1.4	1.2	43.9	< LOD	< LOD	1.48
< LOD	< LOD	< LOD	< LOD	< LOD	< LOD	< LOD	< LOD	< LOD	< LOD	< LOD
< LOD	< LOD	< LOD	< LOD	< LOD	< LOD	< LOD	< LOD	< LOD	< LOD	< LOD

Nitro-Tyr	PEA	Putrescine	SDMA	Serotonin	Spermidine	Spermine	Taurine	alpha-AAA	c4-OH-Pro	t4-OH-Pro
< LOD	< LOD	733928.5714	< LOD	< LOD	505357.1429	136964.2857	11250000	< LOD	< LOD	312500
< LOD	< LOD	891071.4286	< LOD	< LOD	775000	244642.8571	12160714.29	< LOD	< LOD	364285.7143
< LOD	< LOD	861363.6364	< LOD	< LOD	1736363.636	486363.6364	12795454.55	< LOD	< LOD	379545.4545
< LOD	< LOD	856818.1818	< LOD	< LOD	1190909.091	431818.1818	13363636.36	< LOD	< LOD	354545.4545
< LOD	< LOD	360000	< LOD	< LOD	1554285.714	1011428.571	21714285.71	< LOD	< LOD	402857.1429
< LOD	< LOD	357142.8571	< LOD	< LOD	1534285.714	1180000	21514285.71	126571.4286	< LOD	428571.4286
< LOD	< LOD	262500	< LOD	< LOD	1133333.333	737500	15833333.33	< LOD	< LOD	293750
< LOD	< LOD	260416.6667	< LOD	< LOD	1118750	860416.6667	15687500	92291.66667	< LOD	312500
< LOD	< LOD	856250	< LOD	< LOD	1365625	931250	2575000	< LOD	< LOD	4812500
< LOD	< LOD	809375	< LOD	< LOD	1312500	800000	2737500	< LOD	< LOD	5312500
< LOD	< LOD	944827.5862	< LOD	< LOD	2058620.69	968965.5172	2693103.448	< LOD	< LOD	4379310.345
< LOD	< LOD	951724.1379	< LOD	< LOD	1855172.414	724137.931	3000000	< LOD	< LOD	5586206.897
< LOD	< LOD	300000	< LOD	< LOD	528000	742000	2180000	< LOD	< LOD	3280000
< LOD	< LOD	386000	< LOD	< LOD	376000	462000	2620000	< LOD	< LOD	3660000
< LOD	< LOD	456818.1818	< LOD	< LOD	1195454.545	1136363.636	2477272.727	< LOD	< LOD	3681818.182
< LOD	< LOD	354545.4545	< LOD	< LOD	779545.4545	695454.5455	2340909.091	< LOD	< LOD	3227272.727
< LOD	< LOD	1911538.462	< LOD	< LOD	1719230.769	730769.2308	9384615.385	< LOD	< LOD	217692.3077
< LOD	< LOD	2173076.923	< LOD	< LOD	2230769.231	976923.0769	10692307.69	< LOD	< LOD	260000
< LOD	< LOD	1636363.636	< LOD	< LOD	1703030.303	630303.0303	8242424.242	< LOD	< LOD	189090.9091
< LOD	< LOD	1669696.97	< LOD	< LOD	1478787.879	436363.6364	8333333.333	< LOD	< LOD	174848.4848
< LOD	< LOD	1442105.263	< LOD	< LOD	2063157.895	978947.3684	13526315.79	< LOD	< LOD	194736.8421
< LOD	< LOD	1626315.789	< LOD	< LOD	2357894.737	1031578.947	14157894.74	< LOD	< LOD	238947.3684
< LOD	< LOD	1700000	< LOD	< LOD	2594444.444	977777.7778	16444444.44	< LOD	< LOD	273333.3333
< LOD	< LOD	1661111.111	< LOD	< LOD	2405555.556	888888.8889	17111111.11	< LOD	< LOD	282222.2222
< LOD	< LOD	476923.0769	< LOD	< LOD	309615.3846	98269.23077	4692307.692	< LOD	< LOD	240384.6154
< LOD	< LOD	473076.9231	< LOD	< LOD	321153.8462	89038.46154	6038461.538	< LOD	< LOD	255769.2308
< LOD	< LOD	110779.2208	< LOD	< LOD	120389.6104	88311.68831	5000000	< LOD	< LOD	177922.0779
< LOD	< LOD	118441.5584	< LOD	< LOD	181818.1818	155844.1558	5701298.701	< LOD	< LOD	192207.7922

Nitro-Tyr	PEA	Putrescine	SDMA	Serotonin	Spermidine	Spermine	Taurine	alpha-AAA	c4-OH-Pro	t4-OH-Pro
		2.27654E-05			0.372782947	0.002557008	0.011415023			0.858949698
		3.68842E-05			0.011085954	0.544030842	0.037084055			0.00180891
		0.130129115			0.025280163	0.055487821	0.001040367			0.225601714
		0.002299597			0.141115087	0.130727779	0.000904252			0.008997136
		0.009748044			0.002894346	0.004705355	0.006338794			0.025044127

Table S7. Concentration of metabolites used in for metabolic flux analysis. Concentrations are reported as μM / 500 000 cells (Average of time points 0, 10, 30 and 90 min).

	**DU145**			**DU145-RR**			**LNCaP**			**LNCaP-RR**		
	**#1**	**#2**	**#3**	**#1**	**#2**	**#3**	**#1**	**#2**	**#3**	**#1**	**#2**	**#3**
**Citrate**	7.77	7.97	11.35	9.67	3.81	21.32	11.61	9.14	9.42	117.23	19.34	54.70
**Malate**	33.23	27.87	37.77	26.69	17.62	110.53	28.15	27.49	31.27	100.73	27.07	38.34
**Aspartate**	221.72	175.25	310.34	190.91	119.66	933.39	172.03	108.65	159.03	1560.42	331.40	829.06
**Glutamate**	993.99	1003.60	1352.51	932.34	596.93	4112.85	405.97	355.22	527.94	2756.83	705.82	1336.07
**Succinate**	15.72	9.64	15.78	39.01	10.51	66.35	21.48	9.20	9.71	430.59	76.31	251.79

Figure S1. Analysis of Gln metabolism in DU145 RR and LNCaP RR cells. (**A**) Gene expression analysis in parental and RR PCa cells showed similarity in deregulation pattern of genes involved in the control of TCA. (**B**) Absolute log2 fold change of genes involved in TCA cycle regulation. (**C**) Principal component analysis (PCA) to visualize metabolic differences and the relationship of the analyzed parental and radioresistant (RR) cells. The PCA was performed using the changes in metabolic profiles of amino acids and biogenic amines in parental and RR cells. The differences between analyzed cells are driven e.g., by altered levels of the glucogenic amino acids, including Gln and Glu. The variance explained by PC1 = 52.6% and PC2 = 17.3%. (**D**) Schematic of the model used to quantify the metabolic fluxes in DU145 and LNCaP cell lines. [U-$^{13}C_5$]Glutamine was used as the driving function. The quantified reactions were: glutaminase (V_{Glnase}), glutamate consumption other than the TCA cycle (V_{GluOut}), glutamate dilution from sources other than the TCA cycle (V_{GluDil}), aspartate transaminase (V_X), glutamate dehydrogenase (V_{GDH}), oxidative and reductive directions of isocitrate dehydrogenase (V_{IDH} and V_{IDHr}, respectively), the lower portion of the TCA cycle (V_{TCA}), the scrambling of label due to the equilibrium with fumarate (V_{SC}), citrate synthase (V_{CS}) and ATP-dependent citrate lyase (V_{Acly}). (**E**) Metabolic fluxes analysed in LNCaP and DU145 parental and RR cells. Data are mean ± s.d. (**F**) Glutaminase / TCA cycle ratio based on the metabolic flux data. Data are mean ± s.d. *$p < 0.05$; ***$p < 0.001$.

Figure S1

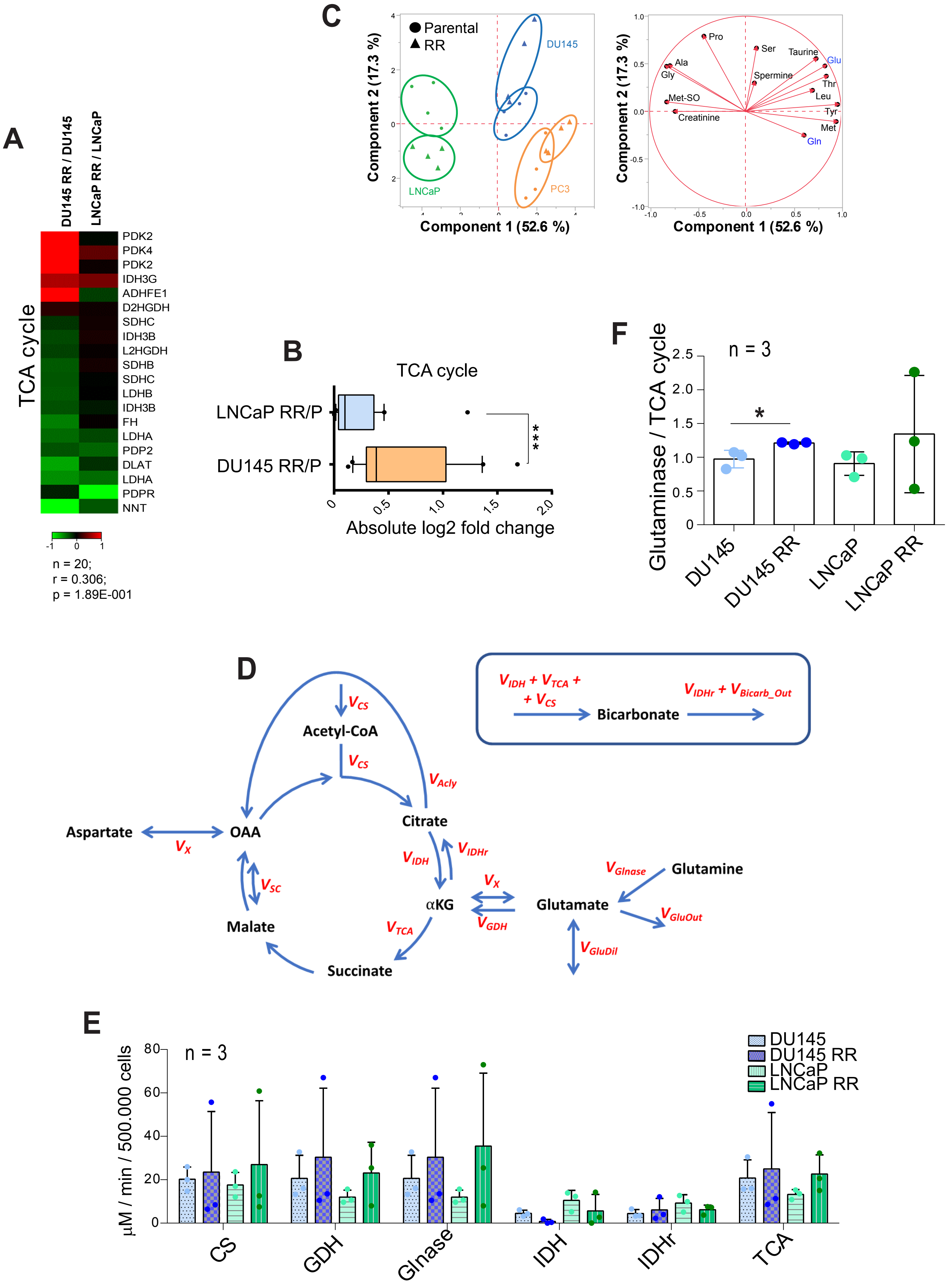

A
DU145 RR / DU145
LNCaP RR / LNCaP
TCA cycle
PDK2
PDK4
PDK2
IDH3G
ADHFE1
D2HGDH
SDHC
IDH3B
L2HGDH
SDHB
SDHC
LDHB
IDH3B
FH
LDHA
PDP2
DLAT
LDHA
PDPR
NNT
-1 0 1
n = 20;
r = 0.306;
p = 1.89E-001
B
TCA cycle
LNCaP RR/P
DU145 RR/P
***
Absolute log2 fold change
C
Parental
RR
DU145
LNCaP
PC3
Component 1 (52.6 %)
Component 2 (17.3 %)
Pro
Ser
Taurine
Glu
Ala
Gly
Spermine
Thr
Leu
Met-SO
Tyr
Creatinine
Met
Gln
D
Acetyl-CoA
Citrate
OAA
Aspartate
Malate
Succinate
αKG
Glutamate
Glutamine
Bicarbonate
E
n = 3
µM / min / 500.000 cells
DU145
DU145 RR
LNCaP
LNCaP RR
CS
GDH
Glnase
IDH
IDHr
TCA
F
n = 3
Glutaminase / TCA cycle
*
DU145
DU145 RR
LNCaP
LNCaP RR

Figure S2. Radiobiological pathways associated with Gln deprivation. (**A**) Western blot analysis of DDR regulations in response to Gln depletion for 72 h and quantification of the Western blot data. Cells grown in presence of glutamine were used as control. Cells were cultured in swapped medium: LNCaP cells were cultured in DMEM, and DU145 cells were cultured in RPMI. The results showed no impact of culture medium on the observed effect as compared to **Figure 2C**. Data are mean ± s.d. (**B**) Ingenuity pathway analysis revealed downregulation of ATM signaling network in DU145 cells in response to Gln starvation. (**C**) Gln starvation lowered intracellular GSH/GSSG ratio in PCa cells. Data are mean ± s.d. (**D**) Gln depletion in DU145 P and RR cells upregulated the hallmark genes of endoplasmic reticulum (ER) stress response. (**E**) qPCR analysis of ALKBH1 and ALKBH3 expression in PCa cells with or without Gln starvation for 24 h. Data are mean ± s.d. (**F**) Plating efficiency of PCa cells incubated with or without Gln depletion for 24 h. Data are mean ± s.d. (**G**) Radiobiological colony-forming assay and plating efficiency of PCa cells grown with or without Gln supplementation in swapped medium: LNCaP cells were cultured in DMEM, and DU145 cells were cultured in RPMI. The effect of Gln starvation can be seen with a greater extent for DU145 cells independent on a culture medium similar to **Figure 2E**. Data are mean ± s.d. (**H**) The radiosensitizing effect of Gln starvation can be partially rescued in DU145 cells by supplementation of α-KG at final concentration of 2 mM for 24 h. Data are mean ± s.d. *$p < 0.05$; **$p < 0.01$; ***$p < 0.001$.

Figure S2

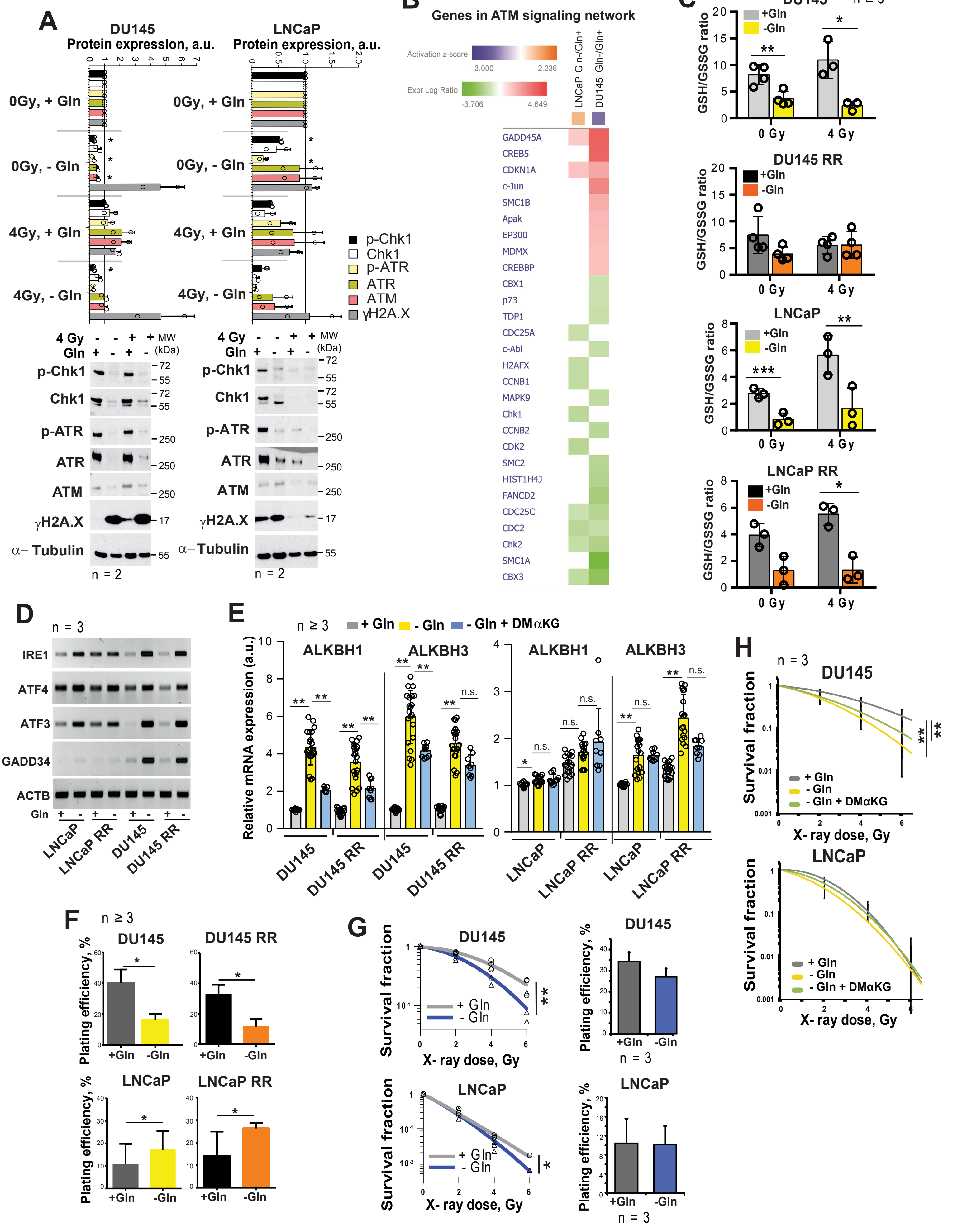
A
DU145
Protein expression, a.u.
LNCaP
Protein expression, a.u.
0Gy, + Gln
0Gy, - Gln
4Gy, + Gln
4Gy, - Gln
p-Chk1
Chk1
p-ATR
ATR
ATM
γH2A.X
4 Gy
Gln
MW (kDa)
α− Tubulin
n = 2
B
Genes in ATM signaling network
Activation z-score
-3.000
2.236
Expr Log Ratio
-3.706
4.649
LNCaP Gln-/Gln+
DU145 Gln-/Gln+
GADD45A
CREB5
CDKN1A
c-Jun
SMC1B
Apak
EP300
MDMX
CREBBP
CBX1
p73
TDP1
CDC25A
c-Abl
H2AFX
CCNB1
MAPK9
Chk1
CCNB2
CDK2
SMC2
HIST1H4J
FANCD2
CDC25C
CDC2
Chk2
SMC1A
CBX3
C
DU145
n ≥ 3
GSH/GSSG ratio
+Gln
-Gln
0 Gy
4 Gy
DU145 RR
LNCaP
LNCaP RR
D
n = 3
IRE1
ATF4
ATF3
GADD34
ACTB
Gln
LNCaP
LNCaP RR
DU145
DU145 RR
E
n ≥ 3
+ Gln
- Gln
- Gln + DMαKG
Relative mRNA expression (a.u.)
ALKBH1
ALKBH3
n.s.
H
n = 3
DU145
Survival fraction
X- ray dose, Gy
LNCaP
F
n ≥ 3
Plating efficiency, %
G
10⁻¹
10⁻²

Figure S3. GLS and MYC expression levels in parental and radioresistant PCa cells and plating efficiency after GLS and MYC knockdown. (**A**) Quantitative real-time PCR (RT-qPCR) analysis of MYC and GLS expression in DU145, DU145 RR, LNCaP and LNCaP RR cell lines. Data are mean ± s.d. (**B**) Plating efficiency of PCa cells in response to siRNA-mediated knockdown of *MYC* or *GLS* gene expression with or without Gln supplementation for 24 h. (**C**) Quantitative real-time PCR (RT-qPCR) analysis of GLS and SLC1A5 expression after MYC knockdown in DU145, DU145 RR, LNCaP and LNCaP RR cells. Data are mean ± s.d. *$p < 0.05$.

Figure S3

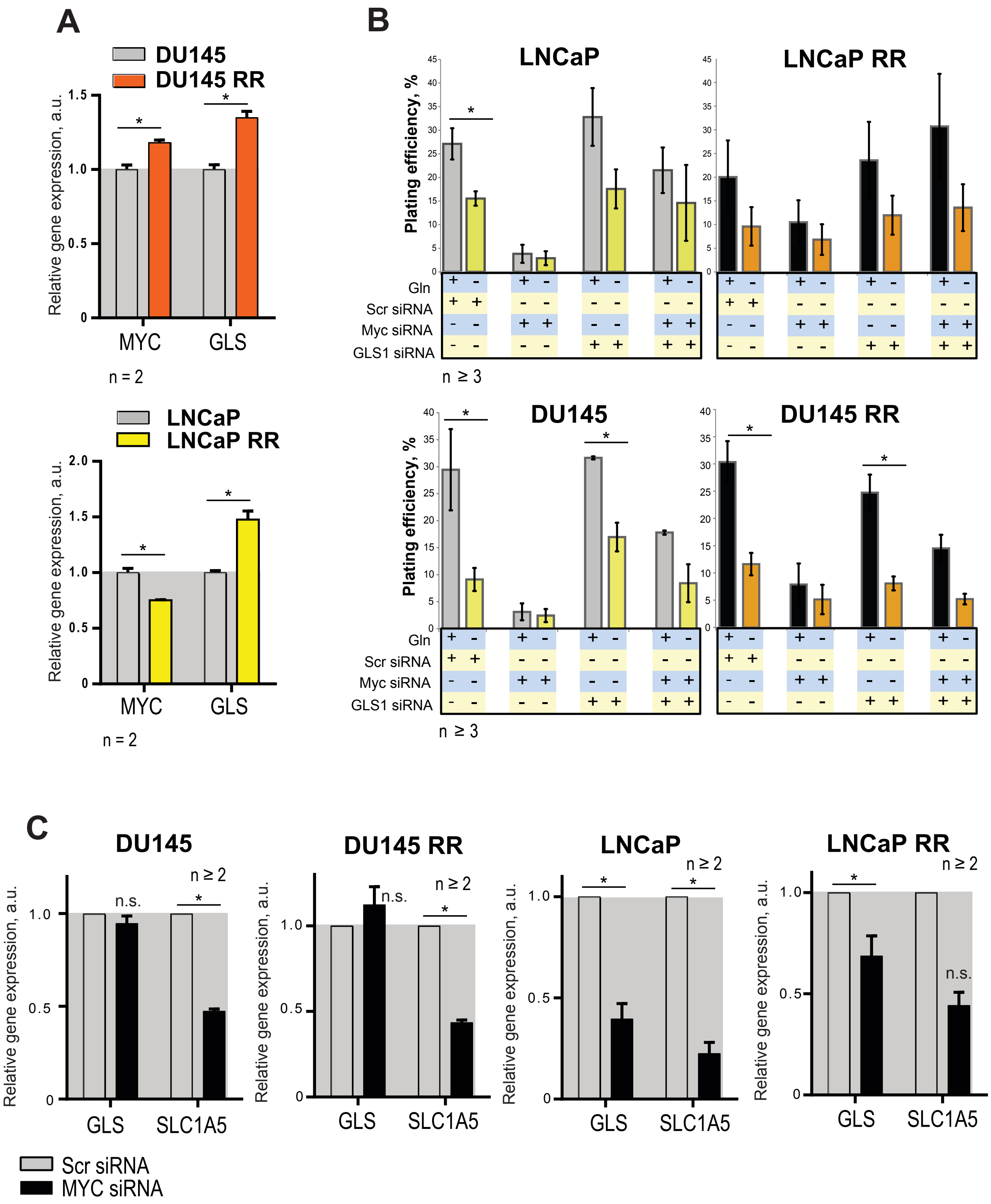
A
DU145
DU145 RR
Relative gene expression, a.u.
MYC
GLS
n = 2
LNCaP
LNCaP RR
n = 2
B
LNCaP
LNCaP RR
Plating efficiency, %
Gln
Scr siRNA
Myc siRNA
GLS1 siRNA
n ≥ 3
DU145
DU145 RR
n ≥ 3
C
DU145
DU145 RR
LNCaP
LNCaP RR
n ≥ 2
n.s.
GLS
SLC1A5
Scr siRNA
MYC siRNA

Figure S4. Chemical inhibition of GLS and MYC in PCa cells. (**A**) Radiobiological colony-forming assay of PCa cells with or without inhibition of MYC. Cells were pretreated with MYC inhibitor 10058-F4 at LD_{50} concentrations for 48 h directly prior clonogenic analysis. Cells were irradiated with indicated X-ray doses directly upon plating. (**B**) Plating efficiency of PCa cells in response to chemical inhibition of MYC, GLS or DMSO treatment. (**C**) DU145, 22Rv1, PC3 and LNCaP were treated for 48 h with different doses of MYC inhibitor 10058-F4 or with GLS inhibitor CB-839 at concentrations ranging from 100 µM to 0 µM and counted with CASY cell counter. LD_{50} values (50% lethal dose) were determined for each cell line individually by non-linear regression analysis using GraphPad Prism software (San Diego, USA). (**D**) Cells were incubated overnight with LD_{50} concentrations of MYC inhibitor 10058-F4. The levels of Glu production were measured to confirm GLS enzymatic activity inhibition. Cells treated with DMSO were used as control. Data are mean ± s.d. (**E**) Radiobiological colony-forming assay of primary PCa and benign prostate hyperplasia (BPH) cultures with or without inhibition of MYC or GLS. Cells were pretreated with MYC inhibitor 10058-F4 or with GLS inhibitor CB-839 at indicated concentrations for 24 h directly prior 3-D clonogenic analysis. Cells treated with DMSO were used as control. (**F**) For primary BPH and tumor cell cultures treated with the MYC inhibitor 10058-F4, the survival fraction values at the different radiation doses were normalized to the values corresponding to the DMSO-treated cells at the same radiation doses. These values for tumor cell cultures and BPH, called radiation enhancement or radiation diminishment ratio depending on the effect caused by chemical inhibitors, were statistically compared using Mann-Whitney test. (**G**) Plating efficiency of patient-derived

primary cultures (PDPC) in response to chemical inhibition of MYC and GLS or DMSO treatment. Data are mean ± s.e.m. *$p < 0.05$; **$p < 0.01$, ***$p < 0.001$, ****$p < 0.0001$.

Figure S4

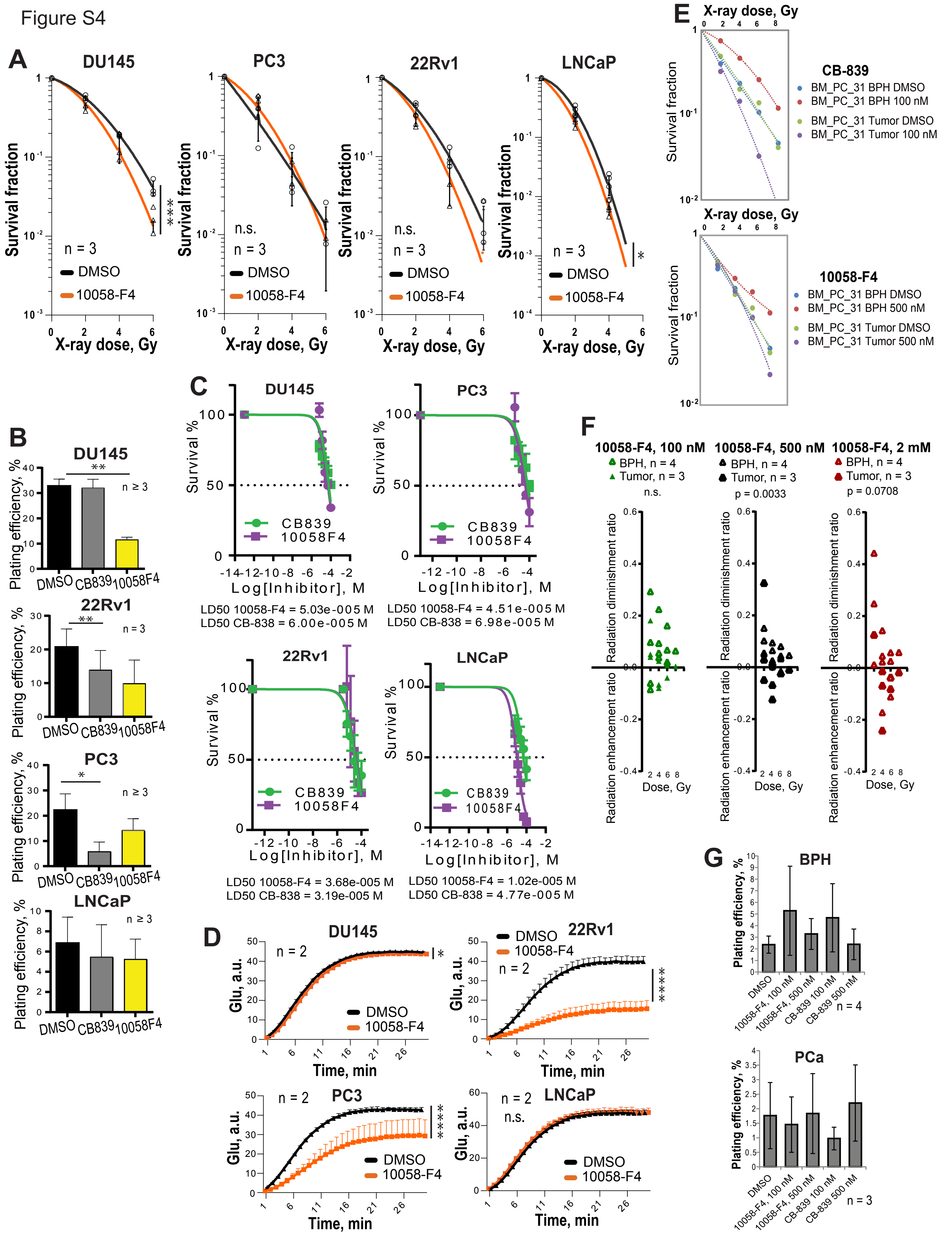

A
DU145
PC3
22Rv1
LNCaP
Survival fraction
X-ray dose, Gy
n = 3
n.s.
DMSO
10058-F4
B
Plating efficiency, %
DMSO
CB839
10058F4
n ≥ 3
n = 3
C
Survival %
Log[Inhibitor], M
CB839
10058F4
LD50 10058-F4 = 5.03e-005 M
LD50 CB-838 = 6.00e-005 M
LD50 10058-F4 = 4.51e-005 M
LD50 CB-838 = 6.98e-005 M
LD50 10058-F4 = 3.68e-005 M
LD50 CB-838 = 3.19e-005 M
LD50 10058-F4 = 1.02e-005 M
LD50 CB-838 = 4.77e-005 M
D
Glu, a.u.
Time, min
n = 2
E
X-ray dose, Gy
CB-839
BM_PC_31 BPH DMSO
BM_PC_31 BPH 100 nM
BM_PC_31 Tumor DMSO
BM_PC_31 Tumor 100 nM
10058-F4
BM_PC_31 BPH DMSO
BM_PC_31 BPH 500 nM
BM_PC_31 Tumor DMSO
BM_PC_31 Tumor 500 nM
F
10058-F4, 100 nM
BPH, n = 4
Tumor, n = 3
n.s.
10058-F4, 500 nM
BPH, n = 4
Tumor, n = 3
p = 0.0033
10058-F4, 2 mM
BPH, n = 4
Tumor, n = 3
p = 0.0708
Radiation diminishment ratio
Radiation enhancement ratio
Dose, Gy
G
BPH
PCa
Plating efficiency, %
DMSO
10058-F4, 100 nM
10058-F4, 500 nM
CB-839 100 nM
CB-839 500 nM
n = 4
n = 3

Figure S5. Mechanisms of PCa radiosensitization in response to Gln starvation. (**A**) The growth-inhibiting effect of Gln deprivation analyzed by MTT assay. Data are mean ± s.d. (**B**) Comparative analysis of gene expression in LNCaP and DU145 parental (P) and radioresistant (RR) cells grown with or without Gln supplementation for 24 h. Gln deprivation results in consistent deregulation pattern of genes involved in the apoptotic mechanisms. (**C**) Absolute log2 fold change of genes involved in apoptosis regulation signature. (**D**) Plating efficiency analyzed after autophagy inhibition in Gln starved cells. Cells were cultured in medium with or without Gln for 24 h. For autophagy inhibitions, cells were then treated with CQ at a concentration of 10 μM for 2 h. (**E**) Plating efficiency of PCa cells in response to *ATG5* knockdown. Cells were transfected with two *ATG5* siRNA or scrambled siRNA for 24 h and then supplemented with fresh media without glutamine for additional 24 h. Data are mean ± s.d. *p < 0.05; **p < 0.01; n.s. – p-value > 0.05.

Figure S5

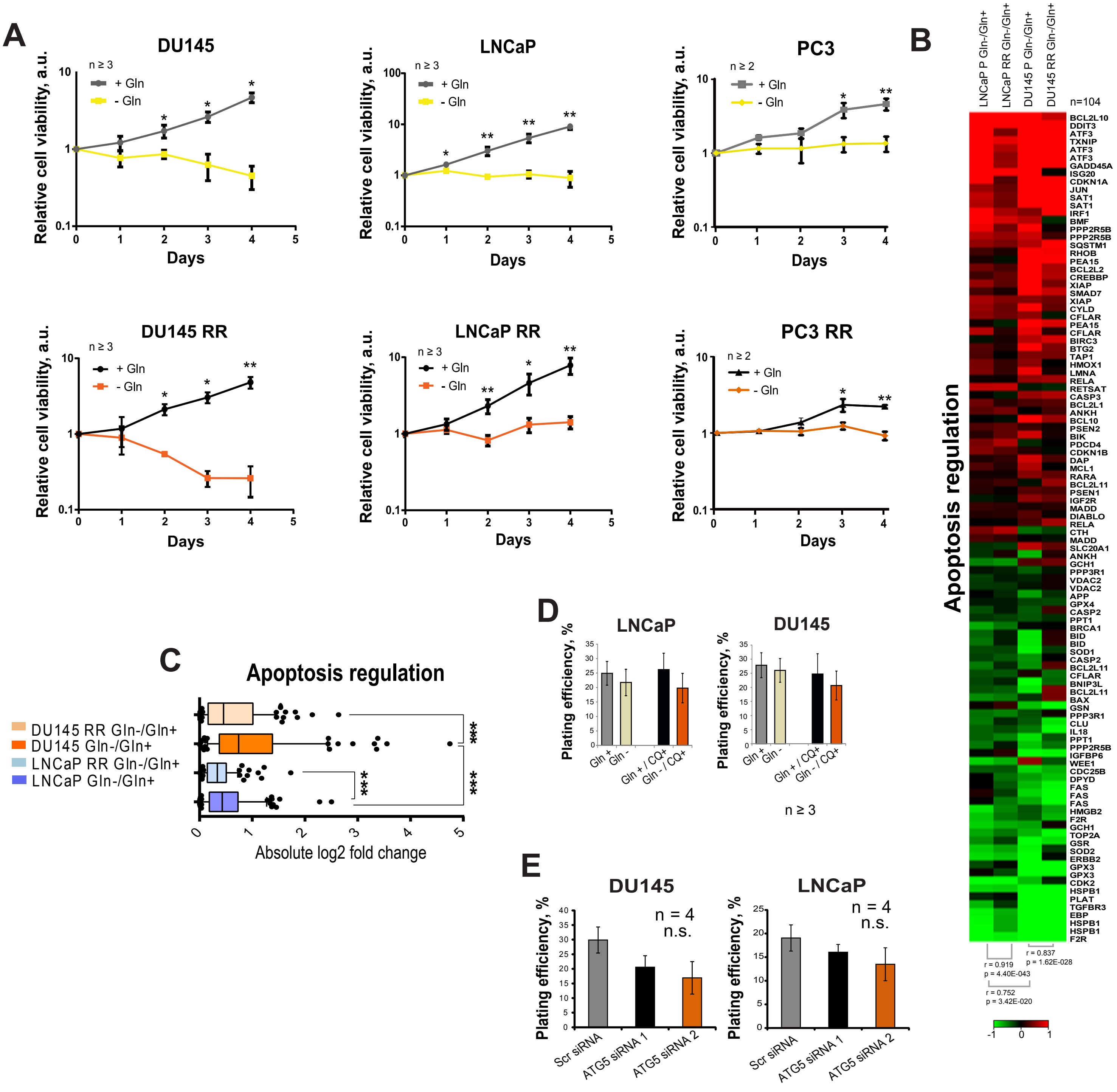

A
DU145
LNCaP
PC3
DU145 RR
LNCaP RR
PC3 RR
Relative cell viability, a.u.
Days
+ Gln
- Gln
n ≥ 3
n ≥ 2
B
LNCaP P Gln-/Gln+
LNCaP RR Gln-/Gln+
DU145 P Gln-/Gln+
DU145 RR Gln-/Gln+
n=104
Apoptosis regulation
r = 0.919
p = 4.40E-043
r = 0.837
p = 1.62E-028
r = 0.752
p = 3.42E-020
C
Apoptosis regulation
DU145 RR Gln-/Gln+
DU145 Gln-/Gln+
LNCaP RR Gln-/Gln+
LNCaP Gln-/Gln+
Absolute log2 fold change
D
LNCaP
DU145
Plating efficiency, %
Gln +
Gln -
Gln + / CQ+
Gln - / CQ+
n ≥ 3
E
DU145
LNCaP
n = 4
n.s.
Scr siRNA
ATG5 siRNA 1
ATG5 siRNA 2

Figure S6. Gln metabolism as a regulator of CSC-related gene expression. (**A**) Gene expression profiling of PCa cells replenished or depleted for Gln for 24 h revealed that Gln starvation induced consistent deregulation pattern of genes involved in the CSC maintenance. (**B**) Western blot analysis of LNCaP and DU145 cells treated with DZNep at a concentration of 1 μmol/L for 72 h. (**C**) PCa cells growing under sphere-forming conditions have deregulation of genes involved in amino acid metabolism.

Figure S6

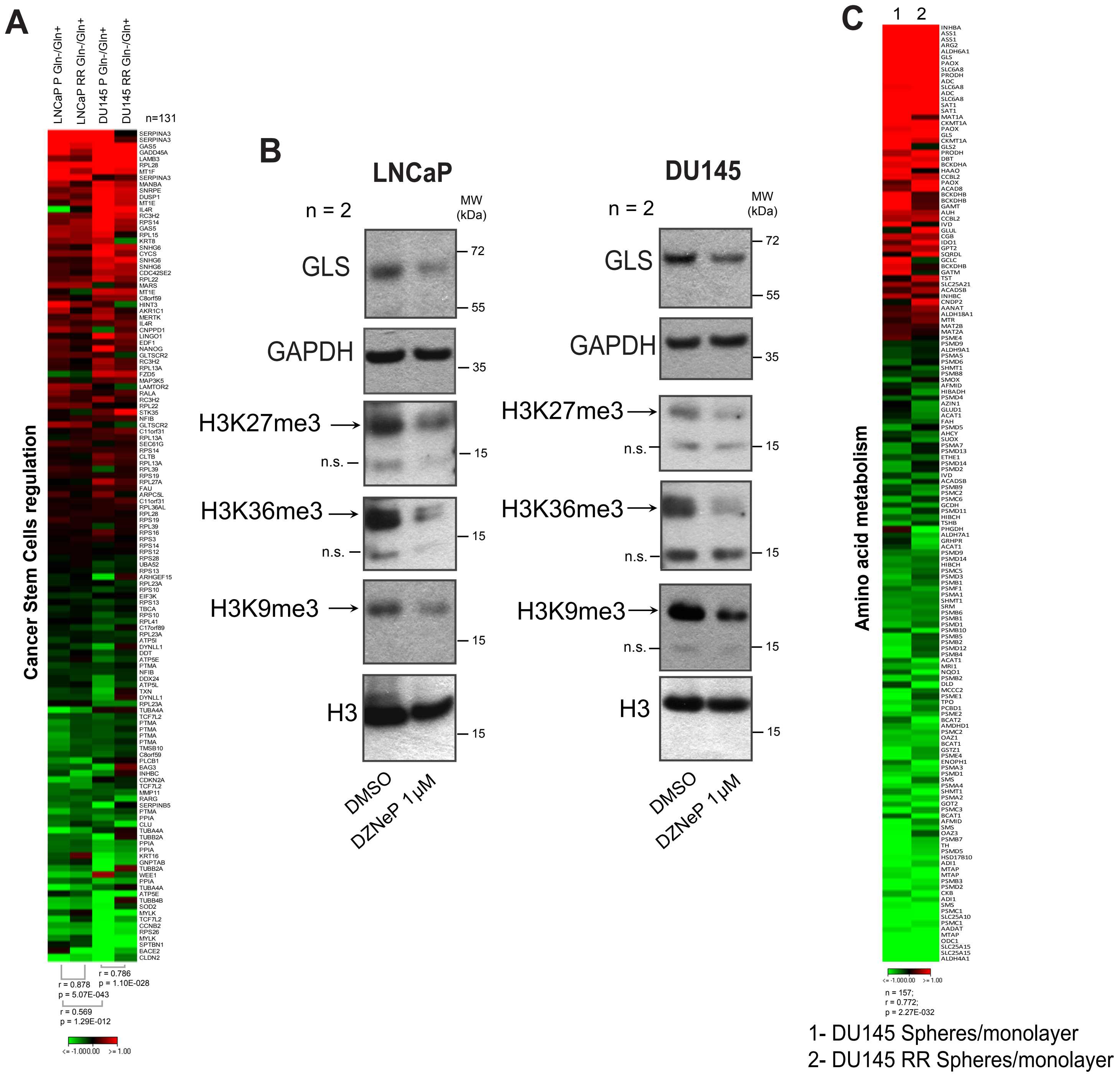
A
LNCaP P Gln-/Gln+
LNCaP RR Gln-/Gln+
DU145 P Gln-/Gln+
DU145 RR Gln-/Gln+
n=131
Cancer Stem Cells regulation
r = 0.878
p = 5.07E-043
r = 0.786
p = 1.10E-028
r = 0.569
p = 1.29E-012
B
LNCaP
DU145
n = 2
n = 2
MW (kDa)
GLS
GAPDH
H3K27me3
H3K36me3
H3K9me3
H3
n.s.
72
55
35
15
DMSO
DZNeP 1 μM
C
1
2
Amino acid metabolism
n = 157;
r = 0.772;
p = 2.27E-032
1- DU145 Spheres/monolayer
2- DU145 RR Spheres/monolayer

Figure S7. Gln metabolism regulates CSC phenotype and properties. (**A**) Gln starvation decreased the total number of formed tumor spheres. Data are mean ± s.d. (**B**) Fluorescence microscopy analysis of ALDH1A1 expression in patient-derived PCa and BPH tissues. MFI - Mean fluorescence intensity. Data are mean ± s.d. (**C**) Supplementation of DU145 cells with a MYC inhibitor 10058-F4 at a concentration of 10 µM for 48 h significantly lowered radiation-induced $ALDH^+$ cell subset. Data are mean ± s.d. (**D**) Analysis of tumor take in NMRI (nu/nu) xenograft models. PCa cells were pre-incubated in the presence or absence of Gln for 72 h, irradiated with 6 Gy of X-rays or sham irradiated, and injected into the mice in Matrigel as 5×10^3 viable cells/implant. *$p < 0.05$; **$p < 0.01$; n.s. – p-value > 0.05.

Figure S7

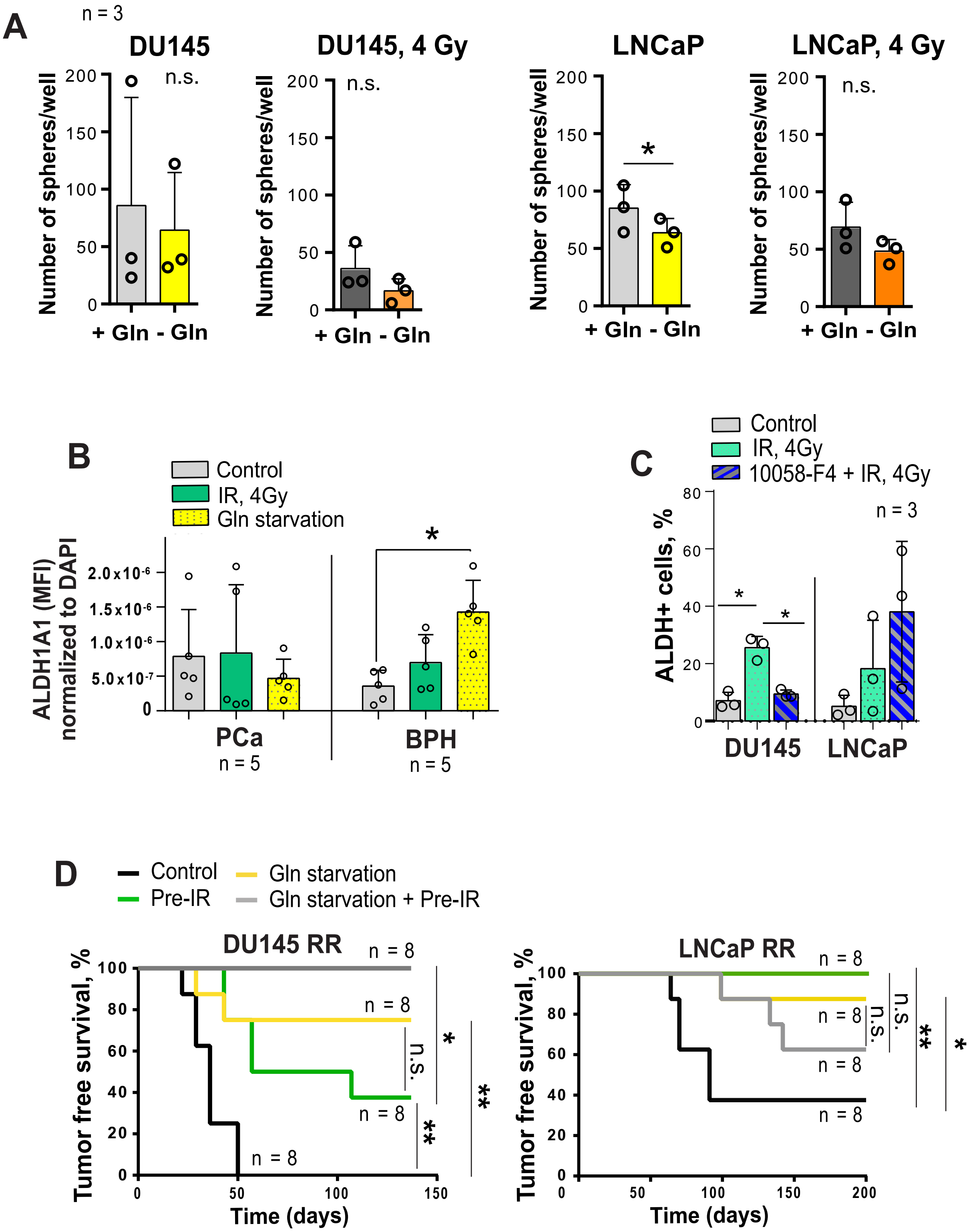
A
n = 3
DU145
DU145, 4 Gy
LNCaP
LNCaP, 4 Gy
Number of spheres/well
n.s.
+ Gln - Gln
B
Control
IR, 4Gy
Gln starvation
ALDH1A1 (MFI) normalized to DAPI
PCa
n = 5
BPH
n = 5
C
Control
IR, 4Gy
10058-F4 + IR, 4Gy
ALDH+ cells, %
n = 3
DU145
LNCaP
D
Control
Pre-IR
Gln starvation
Gln starvation + Pre-IR
DU145 RR
LNCaP RR
Tumor free survival, %
Time (days)
n = 8
n.s.

Figure S8. Analysis of Gln metabolism as clinically relevant biomarker. (**A**) Gene expression of MYC in parental and radioresistant (RR) PCa cells. (**B**) Enrichment of MYC target gene signature in DU145 Gln replenished cells (DU145 Gln+) as compared to DU145 Gln depleted cells (DU145 Gln-); NES – normalized enrichment score; FDR – false discovery rate; p value was determined by random permutation test. (**C**) Analysis of The Cancer Genome Atlas (TCGA) gene expression dataset revealed that high combined expression of MYC and GLS genes is significantly associated with a decrease in relapse-free survival only in PCa patients treated with radiotherapy (n = 56). Median was determined for entire dataset before extraction of 'no RT' and 'RT' groups. Combined expression for GLS + MYC was determined as mean of normalized expression values. A dataset for TCGA prostate adenocarcinoma (PRAD) gene expression was downloaded from UCSC Xena Functional Genomics Explorer: https://xenabrowser.net/.

Figure S8

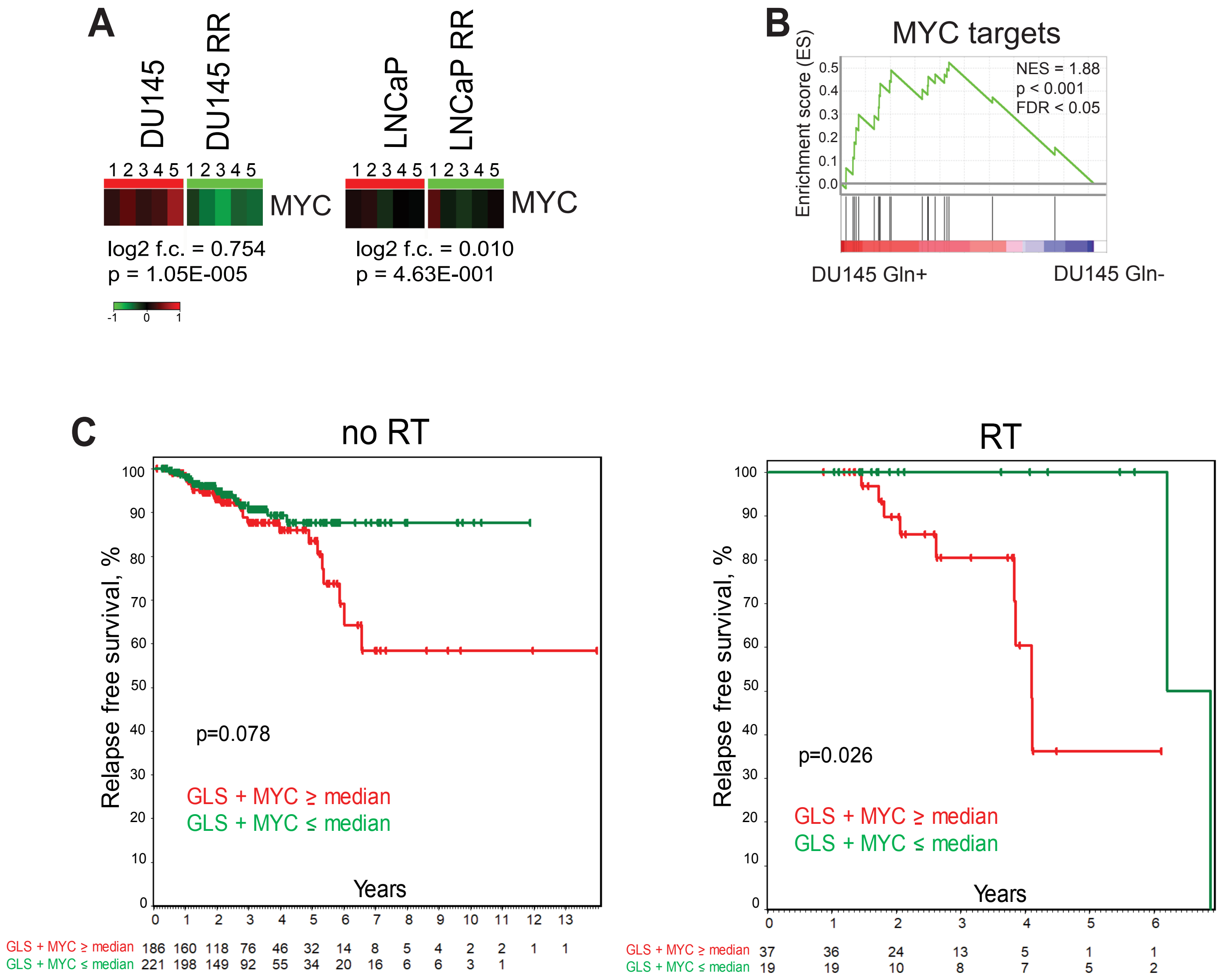
A
DU145
DU145 RR
LNCaP
LNCaP RR
1 2 3 4 5 1 2 3 4 5
MYC
log2 f.c. = 0.754
p = 1.05E-005
1 2 3 4 5 1 2 3 4 5
MYC
log2 f.c. = 0.010
p = 4.63E-001
-1 0 1
B
MYC targets
Enrichment score (ES)
0.5
0.4
0.3
0.2
0.1
0.0
NES = 1.88
p < 0.001
FDR < 0.05
DU145 Gln+
DU145 Gln-
C
no RT
Relapse free survival, %
100
90
80
70
60
50
40
30
20
10
0
p=0.078
GLS + MYC ≥ median
GLS + MYC ≤ median
Years
0 1 2 3 4 5 6 7 8 9 10 11 12 13
GLS + MYC ≥ median 186 160 118 76 46 32 14 8 5 4 2 2 1 1
GLS + MYC ≤ median 221 198 149 92 55 34 20 16 6 6 3 1
RT
Relapse free survival, %
100
90
80
70
60
50
40
30
20
10
0
p=0.026
GLS + MYC ≥ median
GLS + MYC ≤ median
Years
0 1 2 3 4 5 6
GLS + MYC ≥ median 37 36 24 13 5 1 1
GLS + MYC ≤ median 19 19 10 8 7 5 2

Figure S9. Association of GLS and MYC with Gleason score and pathologic T grade. (**A**) Association of GLS and MYC expression with Gleason score in The Cancer Genome Atlas (TCGA) gene expression dataset. Significance calculated by one-way ANOVA followed by Tukey post-hoc analysis. A=Gleason 6, B=Gleason 7, C=Gleason 8, D=Gleason 9. (**B**) Association of GLS and MYC expression with pathologic T stage in The Cancer Genome Atlas (TCGA) gene expression dataset. Significance calculated by one-way ANOVA followed by Tukey post-hoc analysis. A=T2, B=T3, C=T4.

Figure S9

A

GLS

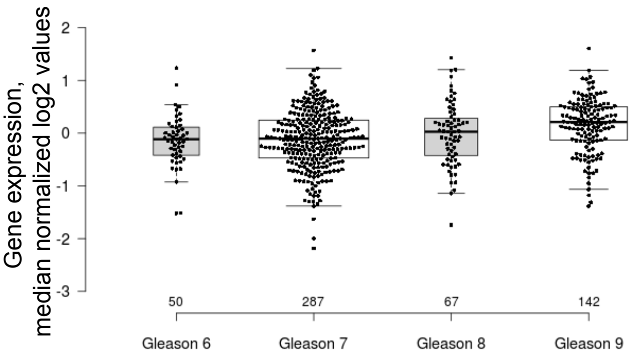

MYC

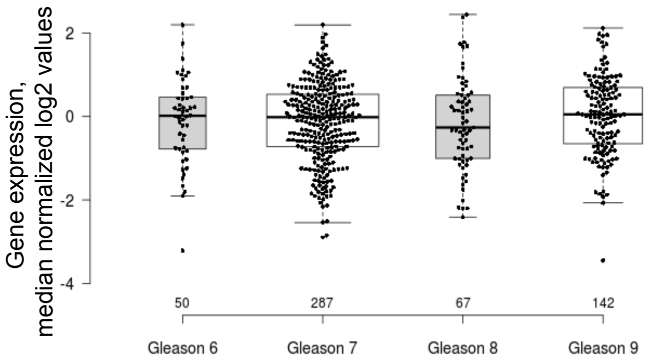

treatments pair	Tukey HSD Q statistic	Tukey HSD p-value	Tukey HSD inferfence
A vs B	0.4230	0.8999947	insignificant
A vs C	1.7965	0.5725111	insignificant
A vs D	5.0594	0.0021338	** p<0.01
B vs C	1.9966	0.4926879	insignificant
B vs D	7.5176	0.0010053	** p<0.01
C vs D	3.3429	0.0855361	insignificant

treatments pair	Tukey HSD Q statistic	Tukey HSD p-value	Tukey HSD inferfence
A vs B	0.1855	0.8999947	insignificant
A vs C	0.5822	0.8999947	insignificant
A vs D	1.1412	0.8323680	insignificant
B vs C	0.5923	0.8999947	insignificant
B vs D	2.1061	0.4461180	insignificant
C vs D	2.0002	0.4911964	insignificant

A=Gleason 6, B=Gleason 7, C=Gleason 8, D=Gleason 9

B

GLS

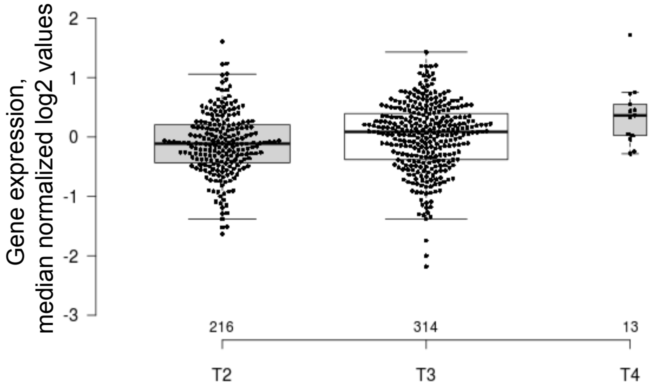

MYC

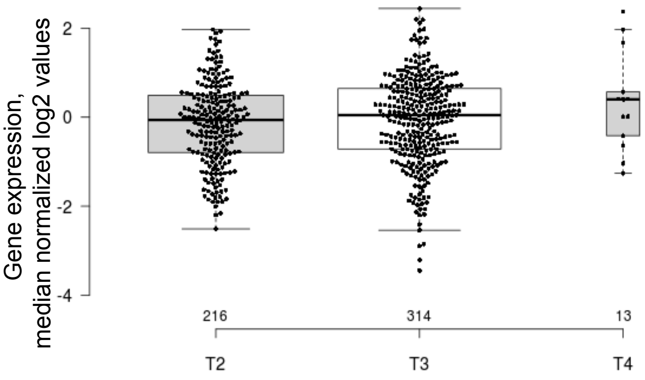

treatments pair	Tukey HSD Q statistic	Tukey HSD p-value	Tukey HSD inferfence
A vs B	3.7858	0.0208790	* p<0.05
A vs C	4.4212	0.0052968	** p<0.01
B vs C	3.2785	0.0541707	insignificant

treatments pair	Tukey HSD Q statistic	Tukey HSD p-value	Tukey HSD inferfence
A vs B	1.3254	0.6077610	insignificant
A vs C	2.5281	0.1749215	insignificant
B vs C	2.1368	0.2870169	insignificant

A=T2, B=T3, C=T4

Figure S10. The radiosensitizing effect of metformin on prostate cancer cells. (**A**) Radiobiological colony-forming assay of DU145 and LNCaP cells with or without glutamine (Gln) starvation and metformin treatment. Cells were pretreated with metformin at a concentration of 2 µM or 200 µM with or without glutamine for 24 h directly before clonogenic analysis. Cells were irradiated with indicated X-ray doses directly upon plating. Data are mean ± s.d; n.s. – non significant. (**B**) Plating efficiency of PCa cells in response to Gln starvation and metformin treatment. Data are mean with s.d; ** $p < 0.01$.

Figure S10

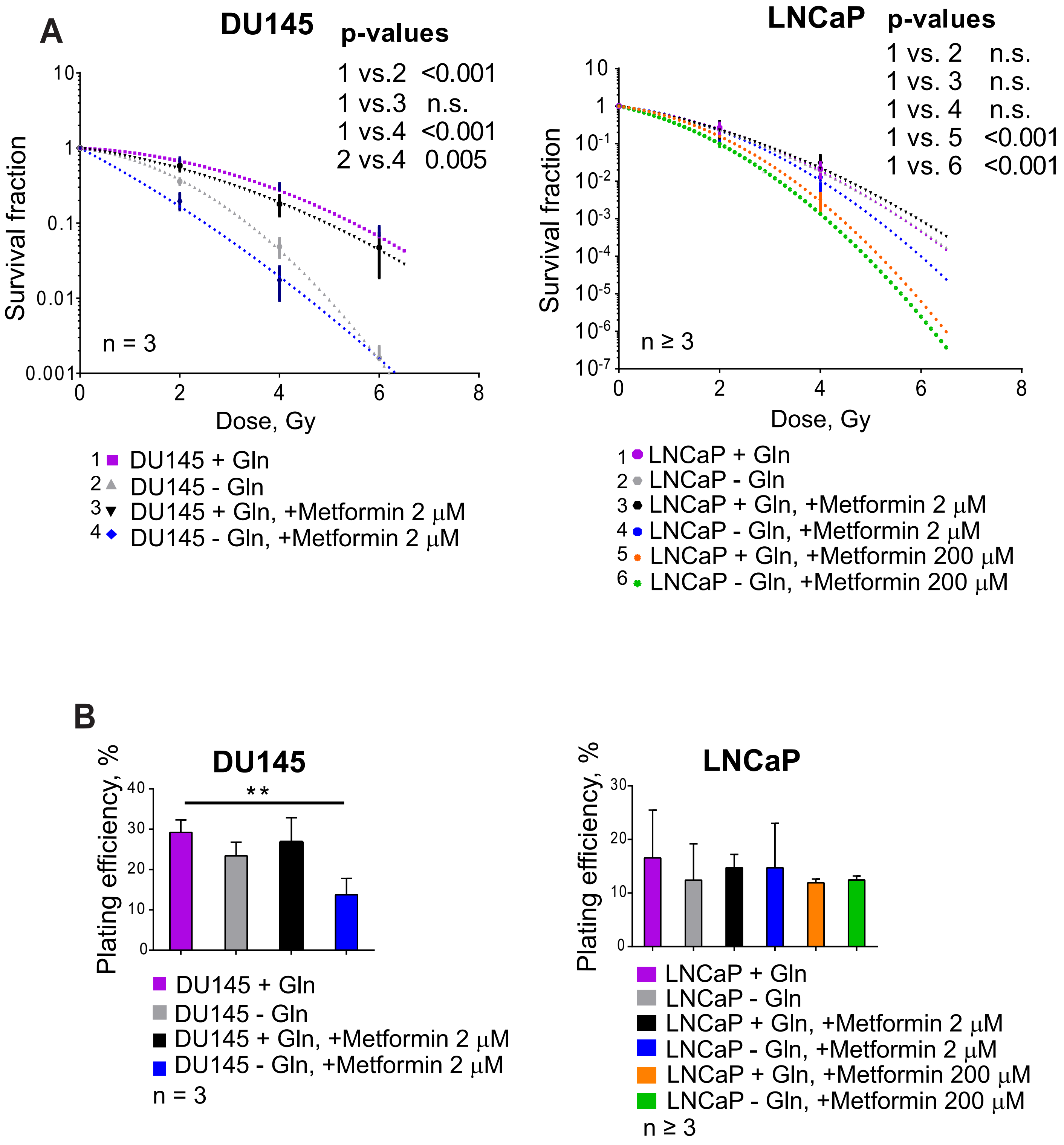
A
DU145
p-values
1 vs.2 <0.001
1 vs.3 n.s.
1 vs.4 <0.001
2 vs.4 0.005
Survival fraction
10
1
0.1
0.01
0.001
0
2
4
6
8
n = 3
Dose, Gy
1 DU145 + Gln
2 DU145 - Gln
3 DU145 + Gln, +Metformin 2 μM
4 DU145 - Gln, +Metformin 2 μM
LNCaP
p-values
1 vs. 2 n.s.
1 vs. 3 n.s.
1 vs. 4 n.s.
1 vs. 5 <0.001
1 vs. 6 <0.001
Survival fraction
10
1
10-1
10-2
10-3
10-4
10-5
10-6
10-7
n ≥ 3
Dose, Gy
1 LNCaP + Gln
2 LNCaP - Gln
3 LNCaP + Gln, +Metformin 2 μM
4 LNCaP - Gln, +Metformin 2 μM
5 LNCaP + Gln, +Metformin 200 μM
6 LNCaP - Gln, +Metformin 200 μM
B
DU145
Plating efficiency, %
40
30
20
10
0
**
DU145 + Gln
DU145 - Gln
DU145 + Gln, +Metformin 2 μM
DU145 - Gln, +Metformin 2 μM
n = 3
LNCaP
Plating efficiency, %
30
20
10
0
LNCaP + Gln
LNCaP - Gln
LNCaP + Gln, +Metformin 2 μM
LNCaP - Gln, +Metformin 2 μM
LNCaP + Gln, +Metformin 200 μM
LNCaP - Gln, +Metformin 200 μM
n ≥ 3
